# SFRP2^+^ fibroblasts orchestrate post-chemotherapy remodeling and define a tumor-driven prognostic program, NBTRP, in adrenal neuroblastoma

**DOI:** 10.1038/s41419-026-09132-y

**Published:** 2026-08-01

**Authors:** Jun Wu, Jian Gu, Guodong Li, Ye Xu, Weihua Li, Yuanxing Liao, Chi Li, Wei Zhang, Dao Wang, Lichun Xie

**Affiliations:** 1 Department of Obstetrics and Gynecology, Department of Paediatrics, Guangdong Provincial Key Laboratory of Major Obstetric Diseases, Guangdong Provincial Clinical Research Center for Obstetrics and Gynecology, Guangdong-Hong Kong-Macao Greater Bay Area Higher Education Joint Laboratory of Maternal-Fetal Medicine; The Third Affiliated Hospital, Guangzhou Medical University, Guangzhou, China; 2 School of Biomedical Science, Hunan University, Changsha, China; 3 Department of Pediatric Hematology and Oncology, The First Affiliated Hospital of Zhengzhou University, Zhengzhou, China; 4 Institute for Brain Research and Rehabilitation, South China Normal University, Guangzhou, China

**Keywords:** Paediatric cancer, Translational research

## Abstract

Neuroblastoma (NB) is a highly heterogeneous pediatric cancer in which chemotherapy resistance and poor prognosis are tightly linked to the tumor microenvironment (TME). Yet, how chemotherapy reshapes stromal components, particularly cancer-associated fibroblasts (CAFs), and the clinical implications of these alterations remain unclear. Here, by integrating single-cell, spatial, and bulk RNA-seq data from nine NB datasets, we systematically characterized chemotherapy-induced TME remodeling. Fibroblast subtypes were defined through clustering, stemness estimation, and functional enrichment analyses, revealing SFRP2^+^ inflammatory CAFs (iCAFs) as the dominant fibroblast population enriched after chemotherapy in adrenal NB TME. These SFRP2^+^ iCAFs exhibited high expression of SFRP2, FBLN1, and chemokines CXCL2 and CXCL3, and were found to promote angiogenesis by signaling to endothelial cells through the CCL2/CXCL2/3/8–ACKR1 axis. Building on the chemotherapy-associated gene expression changes in adrenal NB tumor cells, we developed a 6-gene prognostic model, termed NBTRP. The NBTRP robustly stratified patient survival across multiple cohorts and was associated with reduced cytotoxic T cell infiltration, increased tumor purity, and distinct drug sensitivity profiles. High NBTRP scores predicted enhanced sensitivity to chemotherapeutic agents such as vinblastine and etoposide, as well as improved response to anti–PD-L1 immunotherapy. Functional validation further identified SERPINF1, the top NBTRP feature, as a key effector that promoted NB cell invasion in vitro and modulated drug responses to vincristine, etoposide, cisplatin and cyclophosphamide. Together, our findings uncover SFRP2^+^ iCAFs as pivotal mediators of post-chemotherapy TME remodeling and establish NBTRP and SERPINF1 as clinically relevant biomarkers that bridge tumor–stroma dynamics with prognosis and therapeutic guidance in adrenal neuroblastoma.

## Introduction

Neuroblastoma (NB) is a common extracranial solid tumor of childhood that originates from neural crest cells and predominantly affects the sympathetic nervous system [1–3]. NB, which accounts for approximately 15% of all pediatric cancer-related deaths [3], exhibits striking clinical heterogeneity, with outcomes ranging from spontaneous regression to aggressive metastatic disease [4, 5]. Moreover, the immunosuppressive tumor microenvironment (TME) and inherently low mutational burden hinder the efficacy of current immunotherapies [6, 7], highlighting the urgent need for precision medicine approaches to improve therapeutic outcomes.

The TME plays a critical role as an ecological niche during tumor initiation and progression [5, 7]. The NB TME is composed of stromal cells, immune cells, and Schwann cells, collectively orchestrating tumor behavior [8]. Increasing evidence indicates that the TME contributes to tumor progression, metastasis, and therapy resistance. Among the TME components, cancer-associated fibroblasts (CAFs) have been shown to coordinate with tumor-associated endothelial cells to promote angiogenesis and tumor proliferation [9, 10]. Moreover, crosstalk between CAFs and tumor-associated macrophages (TAMs) facilitates inflammatory responses and TME maturation [11, 12]. CAFs are emerging as potential therapeutic targets for various cancers, including lung [13] and gastric [14] cancers. However, the roles and subtype-specific functions of CAFs within the NB TME remain largely uncharacterized.

With the advancement of single-cell RNA sequencing (scRNA-seq), it has become increasingly instrumental in unraveling cellular heterogeneity in human diseases [15, 16]. Publicly available scRNA-seq datasets of NB now offer unprecedented resolution for dissecting the TME [16–18]. Dong et al. profiled malignant phenotypes and developmental trajectories from embryonic precursors to adrenal NB using scRNA-seq [17]. A recent study mapped the chemotherapy-treated TME landscape of human NB using spatial transcriptomics [19]. Notably, Wienke et al. compared immune cell alterations in pre- and post-chemotherapy NB samples [18]. Collectively, these studies provide a valuable foundation for investigating CAF infiltration and therapy-induced changes within the NB TME.

Despite these advances, the phenotypic plasticity of CAFs and their potential roles in therapy-induced TME remodeling in adrenal NB remain poorly understood. In parallel, chemotherapy may also induce tumor-intrinsic transcriptional rewiring in neuroblastoma cells, yet whether these alterations can inform patient prognosis or therapeutic response remains unclear. To address these gaps, we systematically integrated single-cell, spatial and bulk RNA-seq data from pre- and post-treatment adrenal NB samples. We performed in-depth characterization of CAF subpopulations and their transcriptomic dynamics, and developed a neuroblastoma treatment-related prognosis model (NBTRP) based on chemotherapy-associated tumor-intrinsic transcriptional alterations. This study aimed to characterize the dual impact of chemotherapy on both the TME and tumor cells, uncover functionally relevant stromal alterations, and identify clinically actionable biomarkers for adrenal neuroblastoma.

## Materials and methods

### Data collection and preprocessing

Bulk transcriptomic data and clinical annotations for neuroblastoma (NB) were obtained from two independent cohorts, GSE49710 [20] and E-MTAB-8248 (ArrayExpress) [21]. Clinical information for the TARGET-NB dataset was downloaded from the GTEx Portal, and the associated mRNA expression profiles were retrieved using the SangerBox platform [22]. A summary of the bulk RNA-seq datasets is provided in Supplementary Table 1. For downstream analyses, the expression matrices were normalized using Z-score transformation at the gene level.

scRNA-seq and snRNA-seq data were acquired from GSE137804 [17], GSE218003 [18] and Yu et al. (HTAN Yu et al.) [16]. GSE137804 includes samples from embryonic adrenal (*n* = 2), fetal adrenal (*n* = 4), and adrenal NB (*n* = 11) tissues. GSE218003 comprises adrenal NB samples collected before (*n* = 10) and after (*n* = 14) chemotherapy, including 5 matched pre- and post-treatment patient pairs. The HTAN Yu et al. cohort includes 22 patients with high-risk NB before and after induction chemotherapy. For GSE218003, raw data were processed following the quality control criteria described in the original study [18]. For GSE137804 and HTAN Yu, et al., the processed expression matrix provided by the authors was directly used. Detailed patient information, including clinical characteristics such as COG risk classification, is provided in Supplementary Table 2.

Spatial transcriptomic data were obtained from the study by Slaw, J.T. et al., which was deposited in Zenodo [19]. The dataset included two high-risk adrenal NB patients: NB1 contained paired pre- and post-treatment tissue sections, whereas NB2 included a post-treatment section only. Detailed sample information is provided in Supplementary Table S2. The downloaded spatial transcriptomic data were processed using BayesSpace to enhance gene expression patterns and spatial resolution [23]. Spatial cell type annotations were adopted from the original study [19]. Information regarding the spatial data used for gene expression enhancement is provided in Supplementary Table S3.

### scRNA-seq processing and cell type annotation

Downstream analysis of the scRNA-seq datasets was performed using the Seurat package (v5.2.0) [24]. Low-quality cells were filtered based on standard quality control criteria, including gene count, UMI count, and mitochondrial gene proportion. Data normalization, scaling, dimensionality reduction, and clustering were conducted following the standard Seurat workflow. Batch effects across samples were corrected using Harmony integration [25]. Cell clusters were visualized using UMAP and annotated based on canonical marker genes, with reference to the CellMarker 2.0 database [26] and published NB scRNA-seq studies [17, 18]. Doublets and ambiguous clusters were excluded. Annotated cell types and corresponding marker genes are listed in Supplementary Table 3.

### Spatial transcriptomics analysis

The downloaded spatial transcriptomic data were processed using BayesSpace to enhance gene expression patterns and spatial resolution [23]. Spearman correlation was used to assess the relationship between the enhanced patterns. Spatial cell type annotations were adopted from the original study [19]. Spatial enhancement was performed according to the BayesSpace tutorial and recommended workflow (https://github.com/edward130603/BayesSpace).

### Differential expression and cellular infiltration analysis

Differentially expressed genes (DEGs) among fibroblast subtypes were identified using the FindAllMarkers function in Seurat [24], with thresholds of expression in >25% of cells, log-fold change > 0.25, and *p* < 0.05 (Wilcoxon rank-sum test). The full list of DEGs is provided in Supplementary Table 4. Differences in cellular proportions between pre- and post-treatment groups were assessed using the Wilcoxon rank-sum test for unpaired samples and the paired t-test for matched samples. A two-sided *p* < 0.05 was considered statistically significant.

### Assessment of cell differentiation states

To assess the developmental plasticity and differentiation hierarchy within fibroblast subpopulations, we applied CytoTRACE2 (version 1.1.0) [27]. Log-normalized gene expression matrices were used as inputs. Fibroblast cells were categorized into four phenotypic states (oligopotent, multipotent, unipotent, and fully differentiated) based on CytoTRACE2 scores, enabling downstream analysis of lineage states across conditions.

### Functional enrichment analysis

Functional enrichment analysis was performed using MSigDB gene sets retrieved via the msigdbr package [28]. KEGG pathway enrichment was conducted using the fgsea package [29] based on ranked gene lists from differential expression analyses, with *p* < 0.05 considered significant. HALLMARK pathway activity was assessed using the GSVA package [30] with the ssGSEA method to calculate enrichment scores across fibroblast subtypes.

### Cell–cell communication analysis

Cell–cell communication analysis was performed using the CellChat package (v2.2.0) [31]. Ligand–receptor interactions were inferred based on the CellChatDB human database. Differences in signaling between pre- and post-treatment samples were assessed, focusing on interactions involving SFRP2^+^ fibroblasts and other TME components. Both incoming and outgoing signaling patterns were analyzed, and statistically significant differences were determined using permutation tests (*p* < 0.05).

### Survival analysis

Survival analysis was performed using the survival and survminer packages. Optimal cutoffs for gene expression were determined using the surv_cutpoint function. Kaplan–Meier curves were generated, and differences between groups were assessed using the log-rank test. For cell-type enrichment analysis, GSVA was used to calculate sample-wise scores, followed by survival analysis using the same approach. For prognostic modeling, patients were stratified into high- and low-risk groups based on median risk scores. A two-sided *p* < 0.05 was considered statistically significant.

### XGBoost-based prognostic model, NBTRP, construction and validation

An extreme gradient boosting (XGBoost) model was developed to construct the neuroblastoma treatment-related prognostic signature (NBTRP) based on gene expression data from the GSE49710 cohort. Samples were randomly divided into training (70%) and testing (30%) sets. (1) Feature selection: Differentially expressed genes (DEGs) between pre- and post-treatment samples were identified and intersected with the GSE49710 dataset. Univariate Cox regression analysis was performed to identify prognosis-related genes (BH-adjust-*p* < 0.001). LASSO Cox regression with 10-fold cross-validation was then applied to select the optimal gene set. (2) Model training: The XGBoost model was implemented using the xgboost package (v1.7.8.1) [32] with a Cox proportional hazards objective function. Model parameters were optimized using a grid search strategy. (3) Performance evaluation: Model performance was evaluated using the concordance index (C-index) and time-dependent ROC analysis with AUC values [33]. Internal validation was performed on the test set, and external validation was conducted using the E-MTAB-8248 and TARGET cohorts. (4) Interpretation: Feature importance was assessed based on gain values using the xgboost package.

### TME infiltration analysis

To investigate the association between NBTRP risk scores and stromal or immune cell infiltration within the neuroblastoma TME, three computational frameworks were applied across all three independent cohorts: ESTIMATE [34], CIBERSORT [35], and MCP-counter [36]. Differences between NBTRP high- and low-risk groups were compared using the Wilcoxon rank-sum test (*p* < 0.05).

### Chemotherapeutic sensitivity and immunotherapy response analysis

Chemotherapeutic sensitivity was predicted using the pRRophetic package [37], based on the GSE49710 dataset. The predicted half-maximal inhibitory concentration (IC_50_) values were computed for 138 compounds using the pRRopheticPredict() function with parameters set to tissueType = “allSolidTumors” and dataset = “cgp2014”. Predicted IC50 values were compared between NBTRP risk groups using the Wilcoxon rank-sum test. Detailed drug response scores are provided in Supplementary Table 5.

Immunotherapy response was evaluated using the IMvigor210 cohort. The expression levels of immune checkpoint molecules, including CD274 (PD-L1), CTLA4, PDCD1 (PD-1), and TIGIT, were compared between the NBTRP-high and NBTRP-low groups. Immune checkpoint expression (CD274, CTLA4, PDCD1, and TIGIT) and response rates were compared between groups, and overall survival was assessed using Kaplan–Meier analysis and the log-rank test.

### Multiplex immunofluorescence staining (mIF)

After deparaffinization and dehydration, tissue sections were subjected to antigen retrieval using EDTA buffer (pH 8.0) in a microwave oven for 15 min, followed by natural cooling to room temperature. Sections were then washed three times in PBS (pH 7.4) for 5 min each. Nonspecific binding was blocked with BSA (Solarbio, A8010) for 30 min at room temperature. For the first staining panel, primary antibodies against Enolase (UpingBio, YP-Ab-03855) and SERPINF1 (Proteintech, 26045-1-AP) were applied. For the second panel, primary antibodies against Collagen I (UpingBio, YP-Ab-17694), SFRP2 (Proteintech, 66328-1-Ig), and FBLN1 (Proteintech, 20425-1-AP) were used. Sections were incubated overnight at 4 °C. The next day, slides were washed three times in PBS (5 min each) and briefly dried. Fluorophore-conjugated secondary antibodies corresponding to the host species of the primary antibodies were then applied and incubated for 50 min at room temperature in the dark. After three additional PBS washes (5 min each), nuclei were counterstained with DAPI for 10 min at room temperature and washed three times again in PBS. After that, slides were mounted with an anti-fade mounting medium and stored at 4 °C in the dark. Based on fluorescence intensity and spatial distribution, qualitative, locational, and semi-quantitative analyses of the target proteins were performed.

### Immunohistochemical (IHC) staining

Paraffin-embedded adrenal neuroblastoma tissue sections from patients with different risk stratifications (low-, *n* = 6; intermediate-, *n* = 1; and high-risk, *n* = 9) were collected. Following deparaffinization and rehydration, antigen retrieval was performed. The sections were then incubated with primary antibodies against PEDF (SERPINF1; Proteintech, 26045-1-AP), Fibulin-1 (FBLN1; Proteintech, 20425-1-AP), and SFRP2 (Proteintech, 66328-1-Ig), followed by DAB chromogenic detection and hematoxylin counterstaining. Stained sections were examined under a light microscope, and semi-quantitative scoring was conducted for subsequent analysis.

### SERPINF1 expression analysis in neuroblastoma cell lines

To evaluate the expression of SERPINF1 in NB cell lines, transcriptomic data were obtained from two independent public resources. RNA-seq–based gene expression data for NB cell lines were downloaded from the DepMap Portal (https://depmap.org/portal/), and FPKM-normalized expression data were retrieved from the GSE89413 dataset [38]. For consistency, only the primary NB cell lines were retained for analysis. This resulted in 11 NB cell lines from the DepMap dataset and 4 NB cell lines from GSE89413. In addition, two non-tumor control cell lines, the hTERT immortalized retinal pigmented epithelial cell line, RPE-1, and pooled human fetal brain tissue, were included from GSE89413 as references for baseline expression [38].

### Cell culture and transfection

Human neuroblastoma cell lines CHP212 (Procell Life Science & Technology Co., Ltd., Wuhan, China; Cat. No. CL-0998) and SiMa (Zhejiang Meisen Cell Technology Co., Ltd., China; Cat. No. CTCC-001-1052) were cultured in MEM/F12 medium supplemented with 10% fetal bovine serum (FBS) at 37 °C in a humidified incubator containing 5% CO_2_. For SERPINF1 knockdown, CHP212 cells were transfected with three distinct siRNAs targeting SERPINF1 (si-SERPINF1-1, si-SERPINF1-2, and si-SERPINF1-3), designed and synthesized by Sangon Biotech (Shanghai, China), using Lipofectamine RNAiMAX Transfection Reagent (Thermo Fisher Scientific). The siRNA sequences used are listed in Supplementary Table 6. For SERPINF1 overexpression, the coding sequence (CDS) of human SERPINF1 was synthesized and cloned into the pcDNA3.1(+) vector at NheI/HindIII restriction sites to generate the OE-SERPINF1 construct. The plasmid (OE-SERPINF1) and corresponding empty vector (control) were transfected into SiMa cells using Lipofectamine 3000 Transfection Reagent (Thermo Fisher Scientific). The OE-SERPINF1 sequences are listed in Supplementary Table 6. The insert sequence was verified using Sanger sequencing. Transfection efficiency was confirmed by western blotting.

### Western blot analysis

Total protein was extracted from cultured cells using lysis buffer, and protein concentrations were determined by the BCA assay. Equal amounts of protein were separated by SDS–PAGE and transferred onto PVDF membranes. Membranes were incubated overnight at 4 °C with primary antibodies against SERPINF1 (Proteintech, Cat. No. 26045-1-AP) and GAPDH (loading control), followed by incubation with HRP-conjugated secondary antibodies. The protein bands were visualized using enhanced chemiluminescence (ECL) and imaged with a digital imaging system.

### Drug treatment and cell viability assay

Cells were seeded into 96-well plates at a density of 5 × 10^3^ cells/well and allowed to adhere overnight. The following day, cells were treated with vincristine, etoposide (VP16), cisplatin, or cyclophosphamide (all purchased from Selleck) dissolved in DMSO at the indicated concentration gradients for 48 h. Control wells received an equivalent volume of DMSO. Cell viability was then measured using the Cell Counting Kit-8 (CCK-8) following the manufacturer’s instructions. Dose–response curves and IC_50_ values were calculated using GraphPad Prism software.

### Transwell invasion assay

The cell invasion capacity was assessed using 24-well Transwell chambers (Corning, Cat. No. 353097; membrane pore size: 8 μm). The upper chambers were pre-coated with Matrigel (Corning, Cat. No. 356234), which was diluted 1:10 in serum-free medium. Cells were suspended in serum-free MEM/F12 and seeded into the upper chambers, whereas complete medium containing 10% FBS was added to the lower chambers as a chemoattractant. To better mimic the tumor microenvironment, invasion assays were performed under both normoxic and hypoxic conditions, with hypoxia (1% O₂) used to simulate the tumor microenvironment. After 48 h of incubation, non-invading cells on the upper membrane surface were gently removed using a cotton swab. The invading cells on the lower surface were fixed and stained with crystal violet solution (APExBIO, Cat. No. K1184-100E). The stained cells were imaged and counted under a light microscope in randomly selected fields.

### Statistical analysis

Statistical analyses were performed using R and GraphPad Prism. The Wilcoxon rank-sum test and paired t-test were used for group comparisons as appropriate. Survival differences were assessed using the log-rank test, and categorical variables were compared using Fisher’s exact test. For in vitro experiments, data are presented as mean ± SD from at least three independent experiments. A two-sided *p* < 0.05 was considered statistically significant. In all figures and tables, significance levels are annotated as follows: *p* < 0.05 (*), *p* < 0.01 (**), and *p* < 0.001 or lower (***).

## Results

### Changes in adrenal neuroblastoma and fibroblasts following chemotherapy

**Fig. 1:**
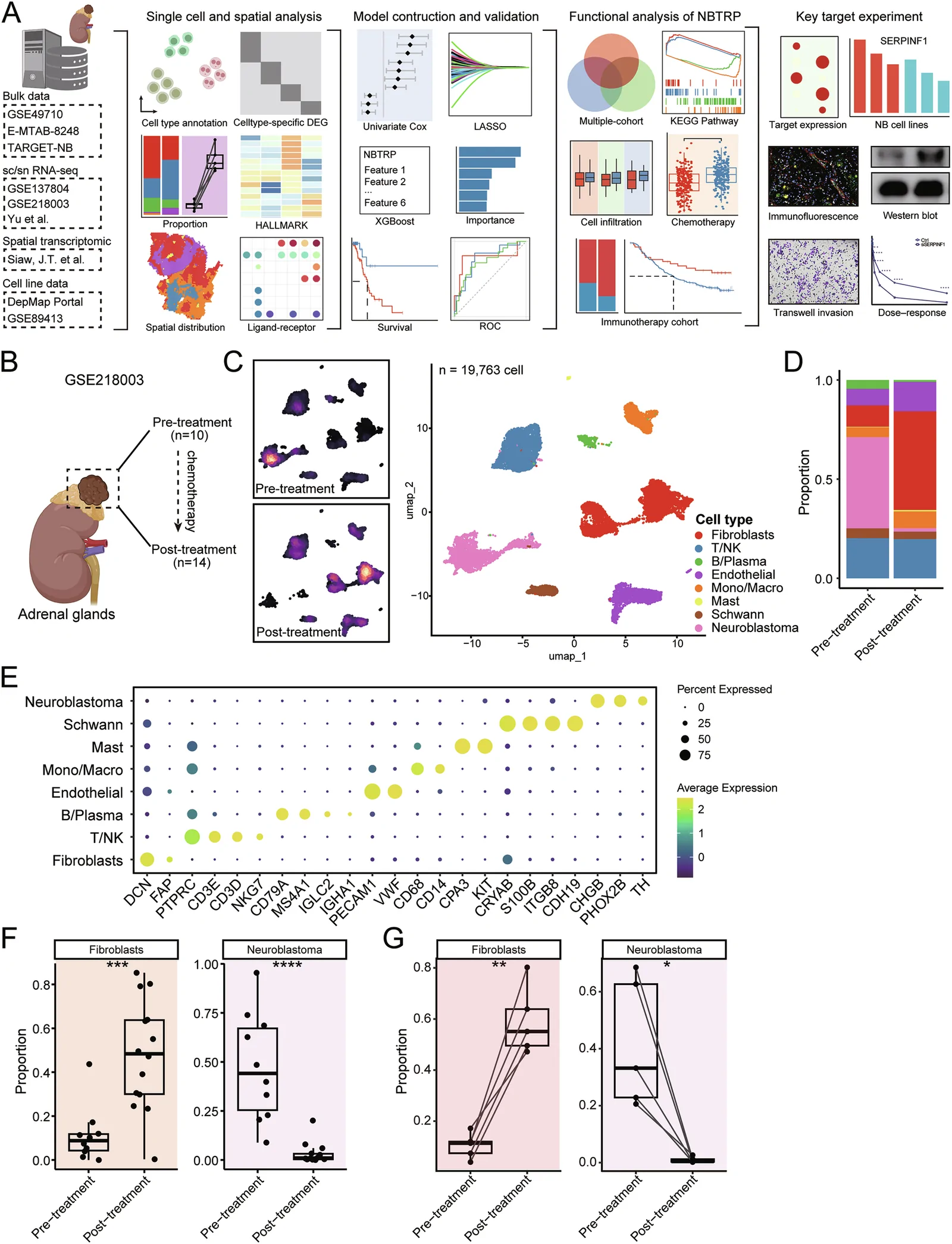
Overview of the study design and single-cell analysis of the chemotherapy cohort. **A** Schematic overview of the study workflow, including data acquisition, analysis, model construction and validation, treatment correlation, and molecular validation. **B** Illustration of the GSE218003 cohort, consisting of 10 pre-treatment and 14 post-treatment neuroblastoma samples. **C** UMAP plot of the single-cell RNA-seq data from all 24 samples in the GSE218003 cohort, showing a cell density map (left) and clustering into 8 major cell types (right). **D** Bar plot comparing the proportions of the 8 major cell types between the pre-treatment and post-treatment groups. **E** Expression patterns of representative marker genes across the 8 major cell types. **F** Box plots comparing the proportions of fibroblasts (left) and NB tumor cells (right) between pre-treatment and post-treatment groups using the Wilcoxon rank-sum test. **G** Paired comparison of fibroblasts (left) and NB tumor cells (right) between matched pre-treatment and post-treatment samples using the paired t-test. Each dot represents one sample in (**F**, **G**).

To systematically characterize the TME remodeling in adrenal NB following chemotherapy, we integrated data from nine datasets, including single-cell, spatial, bulk, and cell line transcriptome profiles. This comprehensive strategy enabled the identification of chemotherapy-associated alterations in TME composition, the construction of a prognostic model via machine learning, and the validation of key targets through functional analyses and molecular experiments (Fig. 1A). We first analyzed the GSE218003 cohort, which includes scRNA-seq data from 10 pre- and 14 post-treatment NB adrenal tumor samples. After quality control (Fig. S1A, B) and unsupervised clustering (Fig. S1C–E), we annotated eight major cell types across 19,763 cells based on canonical markers (Fig. 1C). The proportions of cell type composition differed between pre-treatment and post-treatment samples (Fig. 1D). Each major cell type expressed canonical marker genes, consistent with previous reports, such as PTPRC (also known as CD45) in immune cells, and CHGB, PHOX2B, and TH in NB cells (Fig. 1E). Analysis of cell type proportions across samples revealed a notable increase in fibroblast abundance following chemotherapy (Fig. S2A), which was statistically significant in both the unpaired comparison (Fig. 1F) and the subset of five paired samples (Fig. 1G). In contrast, the proportion of NB cells significantly decreased in post-treatment samples, consistent with prior observations [16, 18] (Fig. 1F, G). No significant changes were observed in the remaining six major cell types (Fig. S2B, C). Our analysis highlights fibroblasts as a major cell population altered after chemotherapy. Given their central roles in the TME, these findings suggest that fibroblasts may critically shape the post-chemotherapy adrenal NB microenvironment and warrant further investigation.

### Functional remodeling of fibroblast subpopulations following chemotherapy

**Fig. 2:**
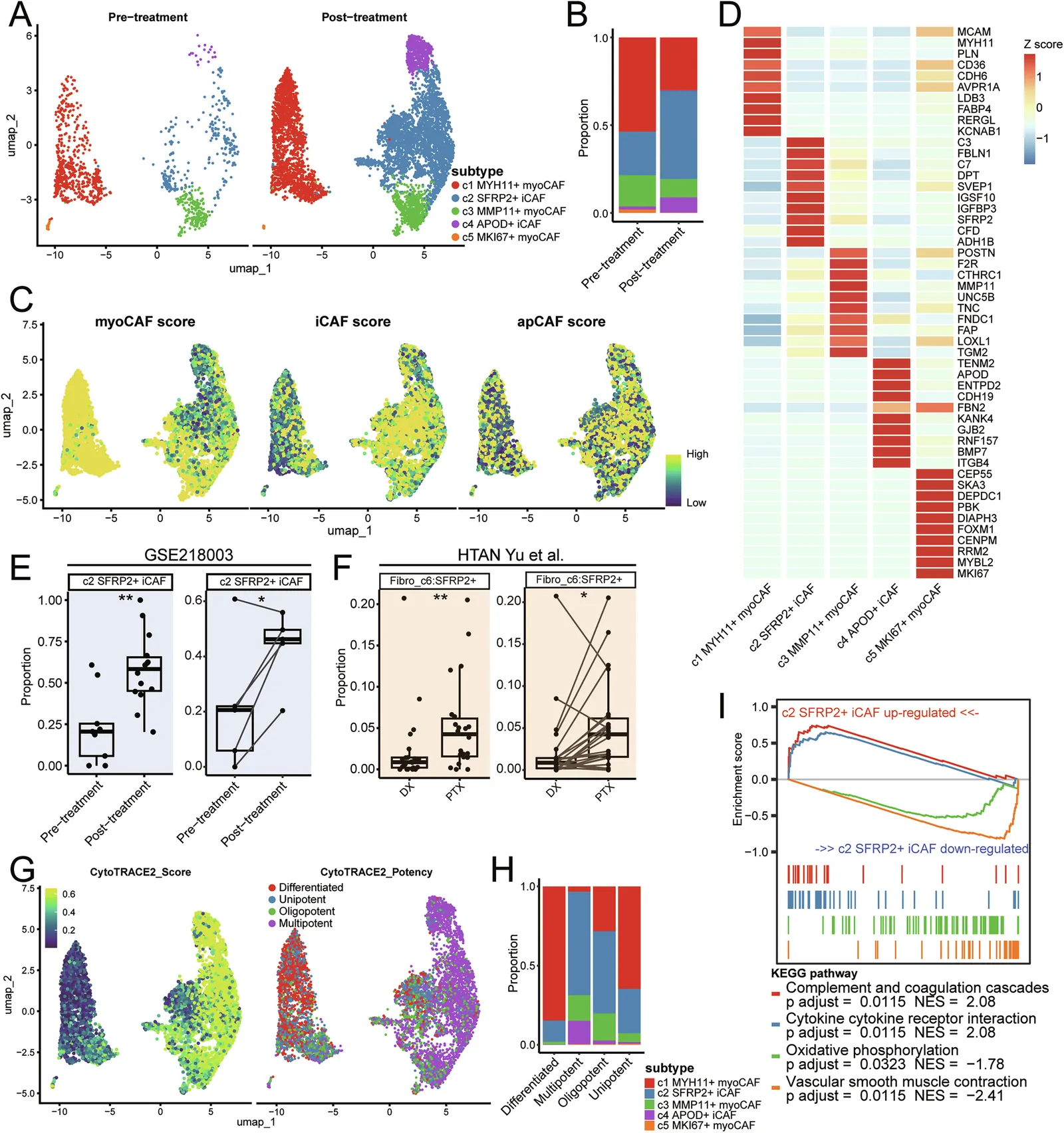
Increased infiltration of SFRP2^+^ iCAFs following chemotherapy in neuroblastoma. **A** UMAP plots showing CAF subclusters from pre-treatment (left) and post-treatment (right) neuroblastoma samples. **B** Bar plot comparing the proportions of five fibroblast subtypes between pre-treatment and post-treatment groups. **C** AddModuleScore-derived scores representing myoCAF, iCAF, and apCAF signatures across CAF subtypes. **D** Heatmap of the top 10 differentially expressed genes across the five CAF subtypes. **E** Boxplots showing differences in the proportions of SFRP2^+^ iCAFs between pre-treatment and post-treatment samples in GSE218003 cohort, assessed using the Wilcoxon rank-sum test (left) and paired t-test (right). **F** Boxplots of proportions of Fibro_c6: SFRP2^+^ based on pre-treatment and post-treatment samples in Yu et al. cohort, evaluated using the Wilcoxon rank-sum test (left) and paired t-test (right). **G** CytoTRACE2 scores across fibroblast cells (left) and corresponding UMAP visualization of four inferred differentiation potency states (right). **H** Bar plot showing the distribution of the five CAF subtypes across the four CytoTRACE-inferred potency states shown in (**G**). **I** Gene Set Enrichment Analysis (GSEA) of KEGG pathways enriched among upregulated and downregulated genes in SFRP2^+^ iCAFs. DX pre-treatment diagnosis, PTX post-induction chemotherapy.

Given the increased abundance of fibroblasts in the post-treatment adrenal NB microenvironment, we performed a systematic analysis of fibroblast heterogeneity and functional states (Fig. 2). Fibroblasts were more abundant in post-treatment samples (Fig. 2A, B). Re-clustering of fibroblasts using Seurat identified six distinct clusters (Fig. S3A). Based on the expression of canonical CAF markers previously reported, the clusters were categorized into three functional subtypes: myofibroblastic CAFs (myoCAFs), inflammatory CAFs (iCAFs), and antigen-presenting CAFs (apCAFs) (Figs. 2C and S3B). Integration of cluster-specific gene expression (Fig. 2D) enabled annotation of five major fibroblast subtypes: c1 MYH11^+^ myoCAF, c2 SFRP2^+^ iCAF, c3 MMP11^+^ myoCAF, c4 APOD^+^ iCAF, c5 MKI67^+^ myoCAF. Among these, c2 SFRP2^+^ iCAF showed statistically significant changes in proportion between pre- and post-treatment samples, in both unpaired and paired analyses (Figs. 2E and S3E, F). We also observed a consistent trend in the Yu et al. cohort across unpaired and paired analyses (Figs. 2F and S4A–C). Notably, c5 MKI67^+^ myoCAFs were reduced after chemotherapy but remained a rare population overall (maximum <8%), whereas c2 SFRP2^+^ iCAFs were more abundant and consistently observed across samples (Fig. 2B and S3E, F). The other three subtypes showed no consistent significant changes in both unpaired and paired analyses (Fig. S3E, F). Given that MKI67 is a proliferation marker, we assessed the differentiation potential of each subtype using CytoTRACE2, which inferred four potency states: differentiated, unipotent, oligopotent, and multipotent (Fig. 2G). c2 SFRP2^+^ iCAFs displayed moderate differentiation potency and were distributed across the intermediate states (Figs. 2H and S3D).

**Fig. 3:**
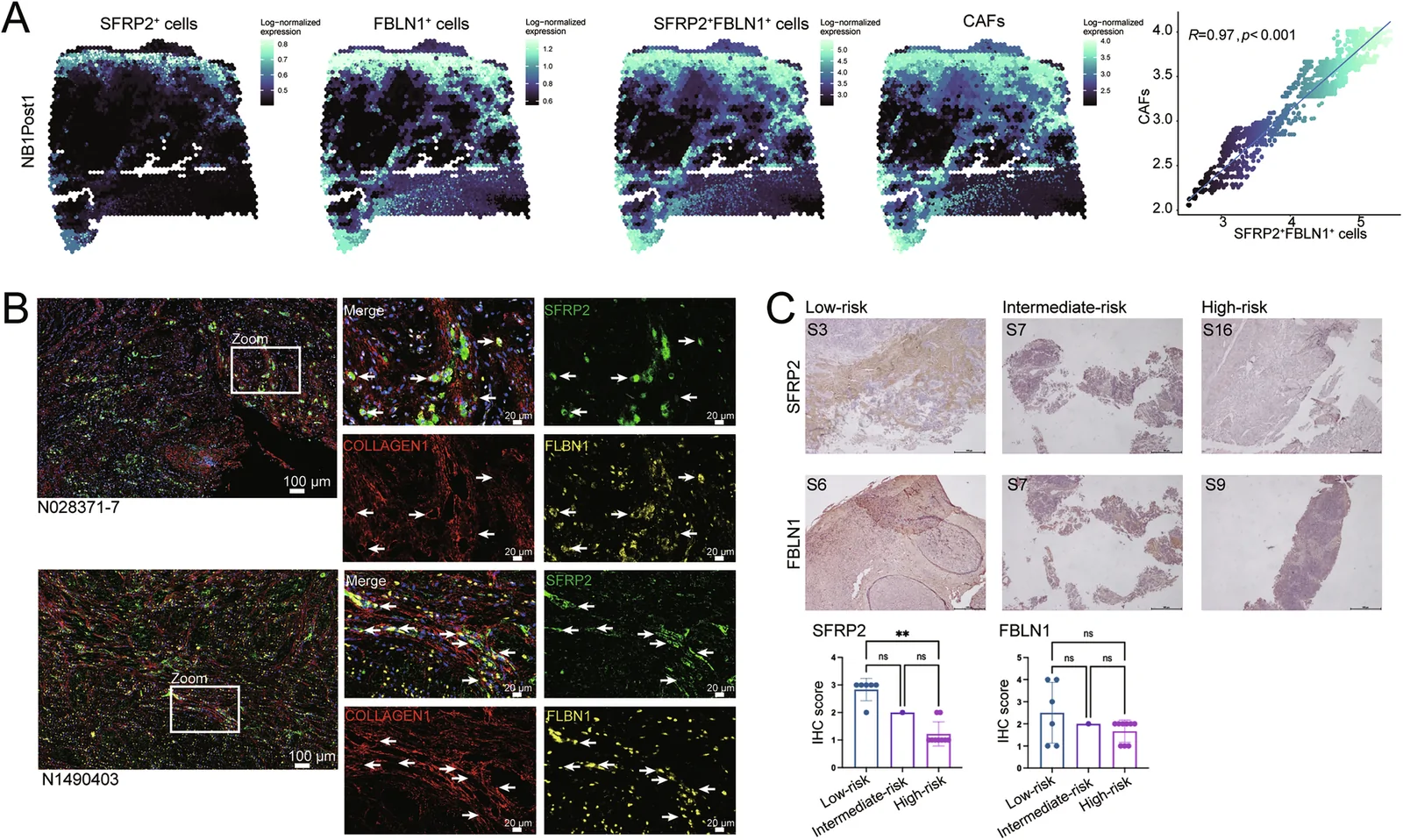
Multiplex immunofluorescence and IHC staining of human neuroblastoma samples. **A** Spatial distribution of SFRP2^+^ cells, FBLN1^+^ cells, SFRP2^+^FBLN1^+^ cells, and CAFs, with Spearman correlation analysis showing spatial association between SFRP2^+^FBLN1^+^ cells and CAFs (right panel). **B** Representative multiplex immunofluorescence panels showing two independent human NB samples. Left: merged images captured at 10× magnification (scale bar, 100 μm). Right: enlarged 40× views of the boxed regions showing merged and individual fluorescence channels for SFRP2 (green), COL1A1 (red), and FBLN1 (yellow; scale bar, 20 μm). White arrows indicate cells exhibiting co-localization of COL1A1, SFRP2, and FBLN1. **C** Representative IHC staining of SFRP2 and FBLN1 in human NB samples across different clinical risk stratifications (low-risk, intermediate-risk, and high-risk groups), along with bar plots showing the semi-quantitative IHC scores among the three groups.

To investigate the functional pathways associated with c2 SFRP2^+^ iCAFs, we performed DEG analysis between c2 and other subtypes. Notably, upregulated genes included FBLN1, SFRP2, complement components (C3, C7), and chemokines such as CXCL3 and CXCL14 (Figs. 2D and S3G). KEGG pathway enrichment analysis (GSEA) revealed that c2 SFRP2^+^ iCAFs were enriched for complement and coagulation cascades (NES = 2.08) and cytokine–cytokine receptor interaction (NES = 2.08), and downregulated for oxidative phosphorylation (NES = –1.78) and vascular smooth muscle contraction (NES = –2.41) (Fig. 2I). Furthermore, spatial transcriptomic analysis revealed that SFRP2^+^FBLN1^+^ cells were spatially co-localized with CAF populations within the post-chemotherapy adrenal NB TME (Figs. 3A and S4D, E). Consistently, mIF staining of three human adrenal NB samples confirmed a subset of cells co-expressing COL1A1, SFRP2 and FBLN1, supporting the presence of c2 SFRP2^+^ iCAFs within the NB TME (Fig. 3B). Notably, IHC staining further showed that SFRP2 expression was significantly higher in low-risk patients compared to high-risk patients (*p* = 0.002), while FBLN1 exhibited a similar trend but did not reach statistical significance (Figs. 3C and S5A, B).

**Fig. 4:**
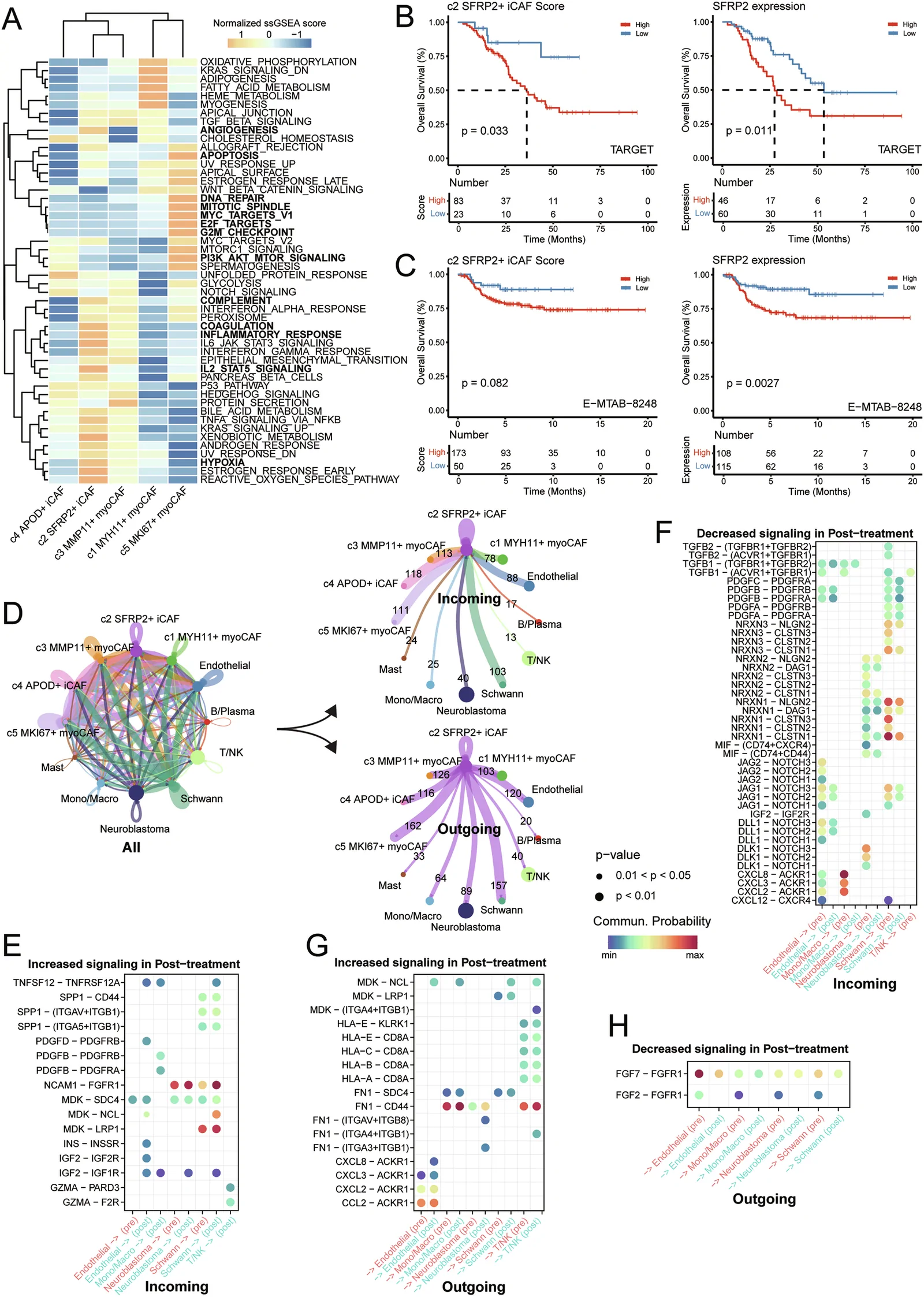
Functional profiling and intercellular communication of SFRP2^+^ iCAFs. **A** Heatmap showing single-sample gene set enrichment analysis (ssGSEA) scores of HALLMARK pathways across the five CAF subtypes. Kaplan–Meier survival curves for SFRP2^+^ iCAF enrichment scores (left) and SFRP2 gene expression (right) in the TARGET (**B**) and E-MTAB-8248 (**C**) neuroblastoma cohorts. Differences in survival were assessed using the log-rank test. **D** Global cell–cell interaction network (left) and incoming/outgoing interaction network centered on SFRP2^+^ iCAFs (right), inferred using CellChat v2. Bubble plots illustrating ligand–receptor interactions from seven major cell types targeting SFRP2^+^ iCAFs that were increased (**E**) or decreased (**F**) in the post-treatment TME. Bubble plots showing ligand–receptor interactions emitted by SFRP2^+^ iCAFs to seven major cell types that were increased (**G**) or decreased (**H**) in post-treatment TME. In bubble plots (**E**–**H**), the bubble size represents interaction strength, and color indicates statistical significance.

Furthermore, GSVA-based analysis of HALLMARK cancer-related pathways showed that c2 SFRP2^+^ iCAFs were enriched in complement and coagulation, angiogenesis, hypoxia, and inflammatory responses (Fig. 4A). c5 MKI67^+^ myoCAFs were enriched for cell cycle–related pathways, including DNA repair, E2F targets, mitotic spindles, G2/M checkpoints, and apoptosis (Fig. 4A). Clinically, high c2 SFRP2^+^ iCAFs GSVA scores were associated with worse overall survival (OS) in NB patients from the TARGET cohort (*p* = 0.033) and showed a trend toward significance in the E-MTAB-8248 cohort (*p* = 0.082) (Fig. 4B, C). High SFRP2 expression was also significantly associated with poor OS in both TARGET (*p* = 0.011) and E-MTAB-8248 (*p* = 0.0027) cohorts (Fig. 4B, C). Moreover, FBLN1, another top marker of c2 SFRP2^+^ iCAFs, showed a similar trend in the TARGET cohort (*p* = 0.08) and strong association within E-MTAB-8248 (*p* < 0.0001) (Fig. S3H, I). Given their abundance and prognostic relevance, we analyzed the intercellular communication patterns of c2 SFRP2^+^ iCAFs.

Based on CellChat analysis (Figs. 4D), c2 SFRP2^+^ iCAFs received the highest number of incoming signals from Schwann cells (*n* = 103), endothelial cells (*n* = 88), and NB tumor cells (*n* = 40), and transmitted outgoing signals to Schwann cells (*n* = 157), endothelial cells (*n* = 120), and NB cells (*n* = 89) (Figs. 4D and S6A, B). To dissect chemotherapy-induced communication changes, we compared incoming and outgoing ligand–receptor interactions between pre- and post-treatment samples (Fig. 4E–H). Post-treatment increased incoming signals, including IGF2–IGF2R from endothelial cells, MDK–SDC4 from multiple sources (NB, endothelial, Schwann), and notably NDK–LRP1, NCAM1–FGFR1, and SPP1–CD44/ITGB1 from Schwann cells (Fig. 4E). Decreased incoming signals included CXCL2/3/8–ACKR1 (Mono/Macro), JAK1–NOTCH1/2/3 and NRXN1/3–CLSTN1/2/3 (Schwann), and DLK1–NOTCH1/2/3, NRXN2–CLSTN1/2/3 (NB) (Fig. 4F). In the outgoing direction, post-treatment increased signals from c2 SFRP2^+^ iCAFs included CCL2–ACKR1 and CXCL2/3/8–ACKR1 (to endothelial cells), FN1–CD44 (to Mono/Macro, NB, and T/NK cells), and HLA-A/B/C/E–CD8A (to NK cells), suggesting enhanced antigen-presentation and stromal–immune crosstalk (Fig. 4G). Conversely, decreased outgoing signals included FGF7–FGFR1 from c2 SFRP2^+^ iCAFs to endothelial cells, Mono/Macro, NB, and Schwann cells (Fig. 4H). Together, these findings highlight c2 SFRP2^+^ iCAFs as the dominant fibroblast subtype that is enriched after chemotherapy. c2 SFRP2^+^ iCAFs may contribute to immunomodulation, angiogenesis, and TME remodeling, making them potential therapeutic targets in post-chemotherapy neuroblastoma. To investigate whether chemotherapy also induces tumor-intrinsic transcriptional alterations in neuroblastoma cells, we further characterized intratumoral heterogeneity and constructed a chemotherapy-associated prognostic model.

### Intratumoral heterogeneity of adrenal neuroblastoma and construction of the NBTRP prognostic model

**Fig. 5:**
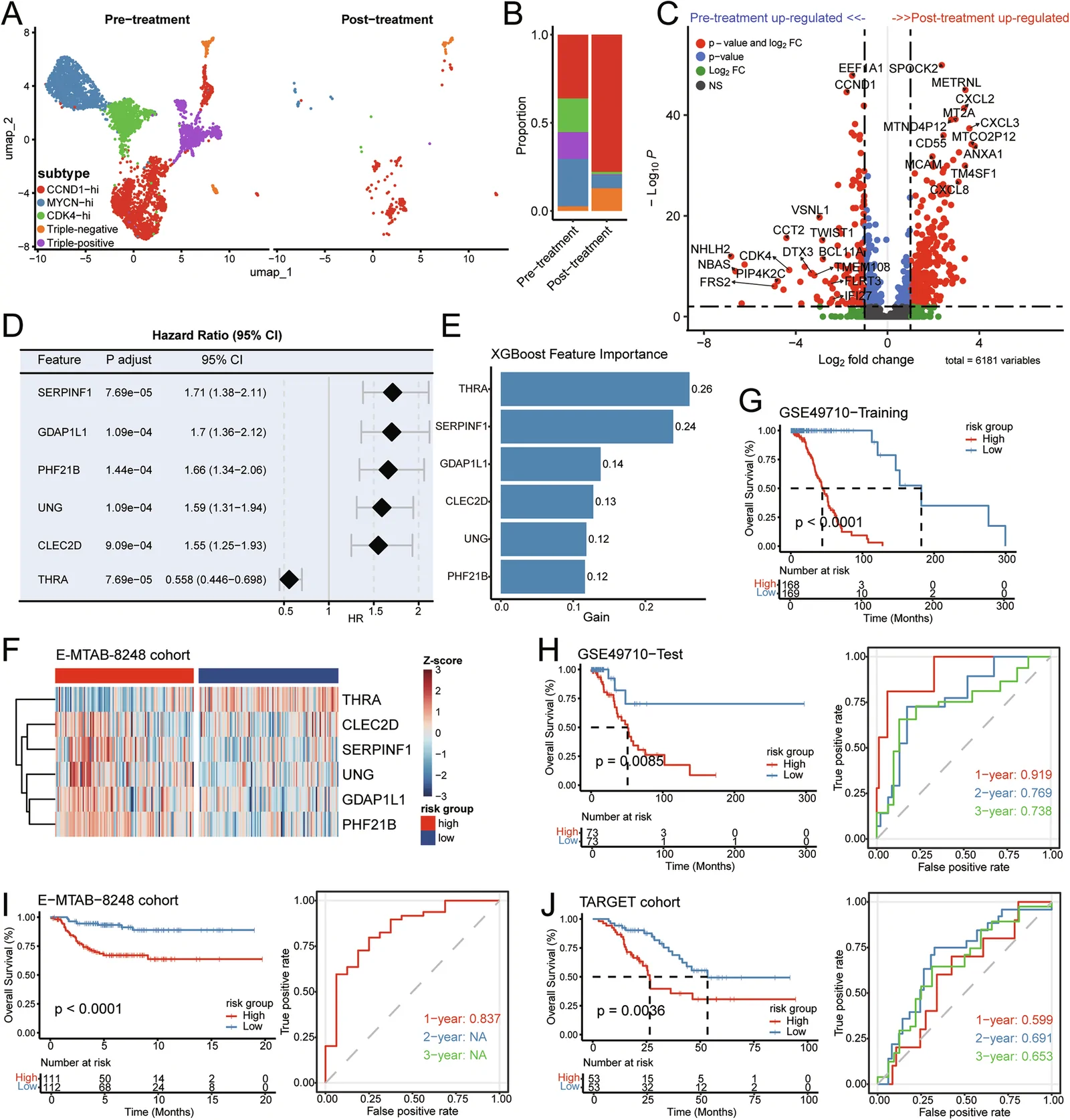
Identification of chemotherapy-associated DEGs and development and validation of the NBTRP prognostic model. **A** UMAP plot showing five NB subtypes identified based on marker gene expression. **B** Bar plot comparing the proportions of the five NB subtypes between pre-treatment and post-treatment samples. **C** Volcano plot of differentially expressed genes (DEGs) between pre-treatment and post-treatment groups. **D** Forest plot showing univariate Cox regression results for the six genes selected by LASSO with non-zero coefficients. **E** Feature importance plot for the six genes in the NBTRP model based on XGBoost, ranked by Gain values. **F** Heatmap showing the expression patterns of the six NBTRP signature genes in the E-MTAB-8248 cohort, stratified by high- and low-risk groups. **G** Kaplan–Meier survival curve of overall survival (OS) based on NBTRP scores in the GSE49710 training cohort. **H** Kaplan–Meier curve (left) and ROC curve (right) of OS prediction in the GSE49710 testing cohort. Kaplan–Meier curves (left) and ROC curves (right) evaluating NBTRP in the external validation cohorts: E-MTAB-8248 (**I**) and TARGET-NBL (**J**). Survival differences in (**G**–**J**) were assessed using the log-rank tests. The area under the curve (AUC) indicates the predictive performance.

To characterize chemotherapy-associated tumor-intrinsic heterogeneity in adrenal NB cells, we performed clustering and annotation of tumor cells from the GSE218003 scRNA-seq dataset. Seven NB clusters were also identified (Fig. S7A) and were annotated into five transcriptional subtypes based on the expression of canonical markers CDK4, CCND1, and MYCN (Fig. S7B), resulting in the following subgroups: CCND1-hi, MYCN-hi, CDK4-hi, triple-positive (CDK4/CCND1MYCN-hi), and triple-negative (Negative) cells (Fig. 5A). A comparison of NB subtype proportions between pre- and post-treatment samples revealed notable heterogeneity in tumor cell infiltration among pre-treatment patients (Figs. 5B and S7C). At the transcriptomic level, NB cells in pre-treatment samples showed elevated expression of CCND1, CDK4, TWIST1, and TMEM108, whereas ANXA1, MCAM, and chemokines CXCL2, CXCL3, and CXCL8 were upregulated after treatment (Fig. 5C). To investigate the prognostic relevance of these chemotherapy-associated tumor-intrinsic DEGs, we conducted univariate cox regression analysis using 70% of samples from the GSE49710 cohort (training set). A total of 32 genes that were significantly associated with overall survival (adjusted *p* < 0.001) were retained. Subsequent LASSO Cox regression identified six genes with non-zero coefficients (Fig. S7D). Among these, five genes were associated with a higher risk (HR > 1), and one gene was protective (HR < 1) (Fig. 5D). These six genes were used as input features to construct an XGBoost-based prognostic model, termed NBTRP. Feature importance was evaluated using the gain metric, with SERPINF1 emerging as the top 2 gene (Fig. 5E). SERPINF1 showed a coefficient (Gain = 0.24) and was associated with significantly high risk (HR = 1.71, adjusted *p* = 7.67e−05) (Fig. 5D, E). Based on the median NBTRP risk score, patients were stratified into high- and low-risk groups. Expression patterns of the six signature genes were consistent with predicted risk (Figs. 5F and S7E, F). In the GSE49710 training set, NBTRP successfully stratified patients with significantly different overall survival (OS) (*p* < 0.0001, Fig. 5G). In the GSE49710 test set, the model remained predictive (*p* = 0.0085), with 1-, 2-, and 3-year AUC values of 0.919, 0.769, and 0.738, respectively (Fig. 5H). NBTRP showed robust prognostic performance in two external validation cohorts. In E-MTAB-8248, NBTRP stratified patients with significant differences in OS (*p* < 0.0001) and achieved a 1-year AUC of 0.837 (Fig. 5I). In the TARGET-NBL cohort, the model also showed significant stratification (*p* = 0.0036), with AUCs of 0.599 (1 year), 0.691 (2 years), and 0.653 (3 years) (Fig. 5J). These results demonstrate that NBTRP is a robust and generalizable prognostic model capable of predicting the survival of patients with neuroblastoma across multiple cohorts and platforms.

### High NBTRP score is associated with proliferation-related pathways and reduced immune infiltration in the neuroblastoma TME

**Fig. 6:**
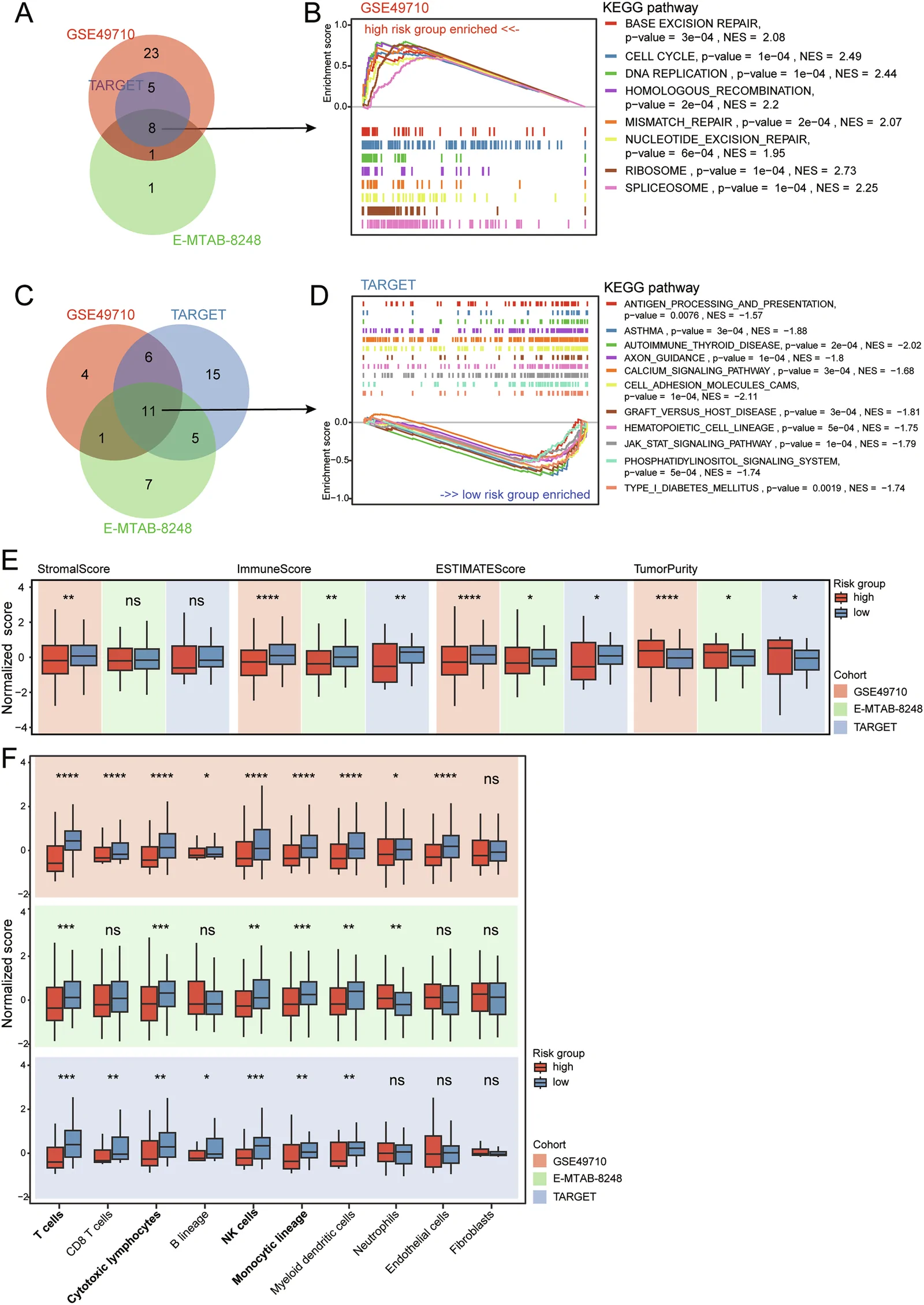
Functional enrichment and immune–stromal landscape associated with NBTRP risk groups. **A** Venn diagram showing the intersection of KEGG pathways enriched in the NBTRP high-risk group across three independent cohorts: GSE49710, TARGET-NBL, and E-MTAB-8248. **B** GSEA plots of the eight overlapping KEGG pathways identified in (A), shown for the GSE49710 cohort. **C** Venn diagram of KEGG pathways enriched in the NBTRP low-risk group across the same three cohorts. **D** GSEA plots of the eleven overlapping KEGG pathways from (**C**), shown for the TARGET cohort. **E** Comparison of four ESTIMATE-derived scores (immune score, stromal score, ESTIMATE score, and tumor purity) between high- and low-risk groups across the three cohorts. Colors indicate cohort identity. **F** Comparison of infiltration scores for 10 immune and stromal cell populations estimated by MCP-counter across the three cohorts. Colors represent cohorts. Statistical significance was assessed using the Wilcoxon signed-rank test.

To explore the biological implications of the NBTRP risk score, we divided the patients into high-risk and low-risk groups based on the median NBTRP score and performed differential expression analysis followed by KEGG pathway enrichment using GSEA. Across three independent cohorts, GSE49710, E-MTAB-8248, and TARGET-NBL, we identified eight overlapping KEGG pathways that were significantly up-regulated in the high-risk group (Fig. 6A). These included key cell proliferation–related pathways, such as cell cycle (NES = 2.49, *p* = 1e-04), DNA replication (NES = 2.44, *p* = 1e-04), mismatch repair (NES = 2.07, *p* = 2e-04), and ribosome (NES = 2.73, *p* = 1e-04) in the GSE49710 cohort (Fig. 6B), with consistent enrichment observed in TARGET (Figu. S8A), and E-MTAB-8248 (Figu. S8B). In contrast, eleven KEGG pathways were consistently upregulated in the low-risk group across the three cohorts (Fig. 6C). These included cell adhesion molecules (NES = –2.11, *p* = 1e-04), antigen processing and presentation (NES = –1.57, *p* = 0.0076), and calcium signaling pathway (NES = –1.68, *p* = 3e-04), with enrichment observed in the TARGET cohort (Fig. 6D), and similarly observed in GSE49710 (Fig. S7C), and E-MTAB-8248 (Fig. S7D).

To further evaluate the relationship between NBTRP risk score and the TME, we analyzed immune and stromal infiltration using multiple computational tools. First, we used CIBERSORT to estimate the proportions of 22 immune cell types across all three cohorts. However, no immune cell population showed consistent and statistically significant differences between high- and low-risk groups across all datasets (Fig. S8E). Next, we used ESTIMATE to compute four scores that reflect TME components. Interestingly, NBTRP score was not correlated with stromal content, but high-risk patients showed significantly lower immune scores than the low-risk group in GSE49710 (*p* < 0.001), E-MTAB-8248 (*p* = 0.0015) and TARGET (*p* = 0.0061) (Fig. 6E). Moreover, tumor purity was significantly higher in the high-risk group in GSE49710 (*p* < 0.001), with consistent trends observed in the other two cohorts. To further clarify these immune differences, we employed MCP-counter, which estimates infiltration of 10 immune and stromal cell populations. Among them, five cell types, T cells, cytotoxic lymphocytes, NK cells, monocytic lineage, and myeloid dendritic cells, consistently showed significantly higher infiltration in the low-risk group across all three datasets (all *p* < 0.05, Fig. 6F).

Together, these results indicate that high-NBTRP tumors are characterized by enhanced proliferative and invasion signaling and reduced immune cell infiltration, reflecting an aggressive and immune-cold phenotype. The overall trends across those cohorts consistently support NBTRP as a functional stratifier of the tumor microenvironment, identifying patients who may benefit from combination therapies targeting both cell cycle progression and immune modulation.

### NBTRP predicts chemotherapy sensitivity and immunotherapy response

**Fig. 7:**
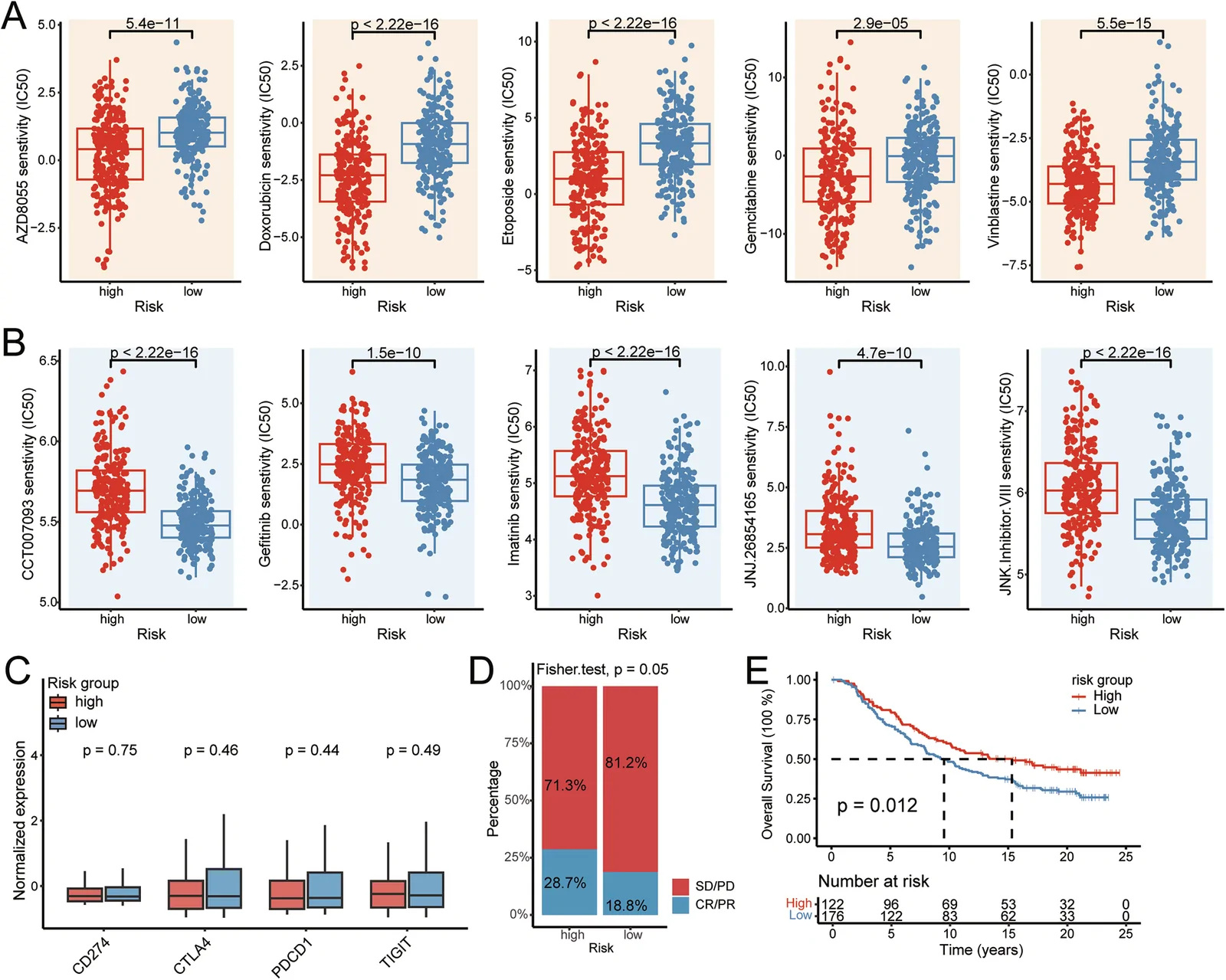
Association of NBTRP with chemotherapy sensitivity and immunotherapy response. **A** Predicted chemotherapeutic agents with lower estimated IC_50_ values in the NBTRP high-risk group compared to the low-risk group, including AAZD8055, Doxorubicin, Etoposide, Gemcitabine, and Vinblastine. **B** Predicted chemotherapeutic agents with lower estimated IC_50_ values in the NBTRP low-risk group, including CCT007093, Gefitinib, Imatinib, JNJ-26854165, and JNK Inhibitor VIII. **C** Expression levels of immune checkpoint genes CD274 (PD-L1), CTLA4, PDCD1 (PD-1), and TIGIT between NBTRP high- and low-risk groups in the IMvigor210 cohort. P-values were calculated using the Wilcoxon rank-sum test. **D** Distribution of clinical responses (responders: CR/PR vs. non-responders: SD/PD) in NBTRP high- and low-risk groups in the IMvigor210 cohort. Significance was assessed using Fisher’s exact test. **E** Kaplan–Meier survival curve comparing overall survival (OS) between NBTRP high- and low-risk groups in the IMvigor210 cohort. Statistical significance was determined using the log-rank test.

Given that NBTRP was derived from chemotherapy-associated transcriptional changes, we next investigated whether it could predict chemotherapeutic drug sensitivity. Among the 138 compounds assessed using the pRRophetic algorithm, IC_50_ values for 114 drugs showed significant differences between NBTRP-defined high- and low-risk groups (Table S5). Of these, 85 drugs had lower predicted IC_50_ values in the high-risk group, and 29 had lower values in the low-risk group. Notably, several clinically relevant agents such as Doxorubicin, Etoposide, Gemcitabine, Vinblastine, and AZD8055 were among the drugs predicted to be more effective (lower IC_50_) in the high-risk group (Fig. 7A). Of these, Doxorubicin, Etoposide, Gemcitabine, and Vinblastine are already used in neuroblastoma treatment and pediatric solid tumor regimens, supporting the clinical relevance of our findings. Conversely, five compounds—CCT007093, Gefitinib, Imatinib, JNJ-26854165, and JNK Inhibitor VIII—were predicted to be more effective in the low-risk group (Fig. 7B). These drugs primarily target tyrosine kinases (e.g., EGFR and BCR-ABL) and stress-activated signaling pathways (e.g., JNK), suggesting distinct vulnerabilities in the low-risk tumors. These results suggest that NBTRP can inform chemotherapy selection and help tailor treatment strategies based on molecular risk profiles.

Given the association between NBTRP and immune cell infiltration, we further evaluated its predictive value in the IMvigor210 cohort, a clinical trial for anti–PD-L1 immunotherapy. PD-L1 (CD274) expression showed no significant difference between the NBTRP high-risk and low-risk groups, and CTLA4, PDCD1 (PD-1), and TIGIT were also not significantly different (Fig. 7C). Importantly, the objective response rate (CR/PR) was 28.7% in the high-risk group higher than 18.8% in the low-risk group (*p* = 0.05, Fisher’s exact test; Fig. 7D). Furthermore, NBTRP stratified patients OS in IMvigor210 patients (*p* = 0.012, log-rank test; Fig. 7E). Taken together, these findings demonstrate that NBTRP is a potential predictive biomarker for both chemotherapy and immunotherapy responses.

### SERPINF1, a key NBTRP feature, is associated with neuroblastoma invasion and chemotherapy response

**Fig. 8:**
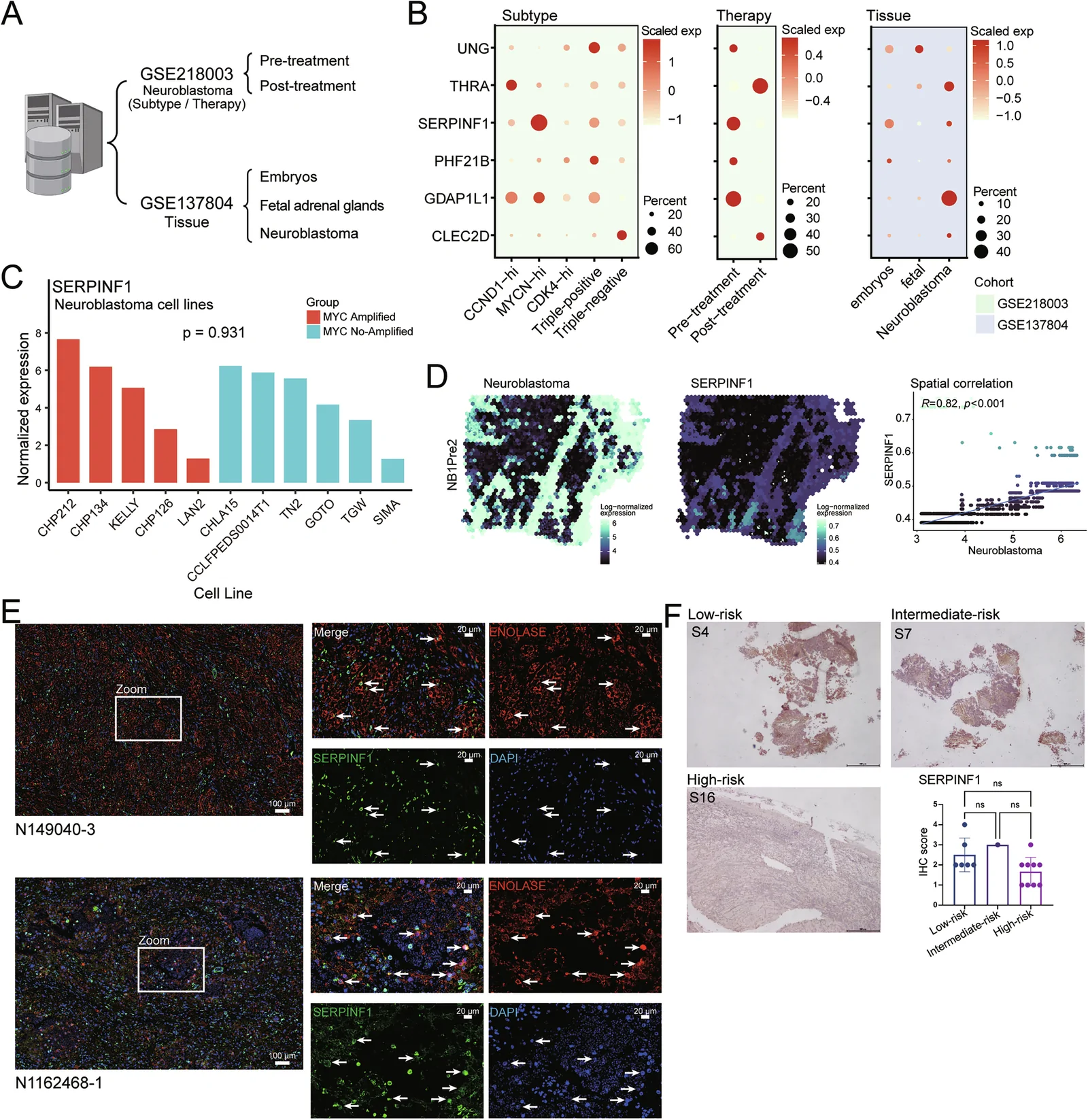
Cross-cohort validation and molecular experimental verification of SERPINF1. **A** Schematic illustration showing that the GSE218003 cohort provides pre- and post-treatment neuroblastoma samples, while the GSE137804 cohort includes three tissue types: embryonic adrenal glands, fetal adrenal glands, and neuroblastoma tissues. **B** Expression patterns of the six NBTRP signature genes across neuroblastoma subtypes (left), treatment types (middle), and tissue types (right). **C** SERPINF1 expression levels in MYCN-amplified (*n* = 5) and non-amplified (*n* = 6) primary neuroblastoma cell lines from the DepMap Portal. P-values were calculated using the Wilcoxon rank-sum test. **D** Spatial distribution of Neuroblastoma and SERPINF1^+^ cells, with Spearman correlation analysis showing spatial association between SERPINF1^+^ cells and Neuroblastoma (right panel). **E** Multiplex immunofluorescence staining of two human NB samples. Left: merged images captured at 10× magnification (scale bar, 100 μm). Right: enlarged 40× views of the boxed regions showing merged and individual fluorescence channels for ENOLASE (red), SERPINF1 (green), and DAPI (blue; scale bar, 20 μm). White arrows indicate cells showing co-localization of ENOLASE, SERPINF1, and DAPI. **F** Representative IHC staining of SERPINF1 in human NB samples across different clinical risk stratifications (low-risk, intermediate-risk, and high-risk groups), along with a bar plot showing the semi-quantitative IHC scores among the three groups.

To further characterize the NBTRP signature, we analyzed its expression across datasets representing treatment status and tissue developmental stages (Fig. 8A). We re-analyzed the GSE137804 scRNA-seq cohort and identified six major cell types, including lymphoid cells, myeloid cells, fibroblasts, Schwann cells, endothelial cells, and NB tumor cells (Fig. S9). Expression profiling of the six NBTRP genes revealed distinct patterns across NB subtypes, treatment states, and tissue origins (Fig. 8B). Notably, SERPINF1 expression was elevated in MYCN-hi NB cells and detectable at lower levels across other subtypes. Other genes showed subtype-specific expression: THRA in the CCND1-hi cluster, and CLEC2D in triple-negative NB cells. When comparing pre- and post-treatment samples, SERPINF1, GDPD1L1, UNG and PHF2B1 were upregulated in pre-treatment samples, whereas THRA and CLEC2D were more highly expressed post-treatment. At the tissue level, SERPINF1 expression was elevated in NB tumor tissues relative to that in fetal adrenal glands, although it was expressed in fewer than 20% of the total cells (Fig. 8B).

SERPINF1 remained among the most important features in the model (Gain = 0.24; Fig. 5E) and showed the strongest prognostic association in univariate analysis (HR = 1.71, adjusted *p* = 7.69e−05; Fig. 5D), leading us to prioritize it for downstream functional validation. SERPINF1 expression was low in human fetal brain tissue but elevated in NB cell lines (Fig. S9F). Moreover, SERPINF1 was detectable in all 11 primary NB cell lines from the DepMap Portal, with no significant difference between the MYCN-amplified and non-amplified lines (*p* = 0.931, Fig. 8C), indicating that its expression is not restricted to a specific genetic background. Spatial transcriptomic analysis further demonstrated a strong spatial co-localization between SERPINF1-positive cells and NB tumor cells within the post-chemotherapy tumor microenvironment (Spearman’s r = 0.82, *p* < 0.001; Fig. 8D). Furthermore, mIF staining of human adrenal NB tissues revealed distinct SERPINF1 expression, with co-localization of SERPINF1 and ENOLASE observed in both examined samples (Fig. 8E). Semi-quantitative IHC scoring revealed detectable SERPINF1 expression across all clinical risk stratifications, although no statistically significant difference was observed among them (Figs. 8F and S10A). Collectively, these results indicate that SERPINF1 is a biologically relevant component of the NBTRP signature and may be involved in adrenal NB biology.

**Fig. 9:**
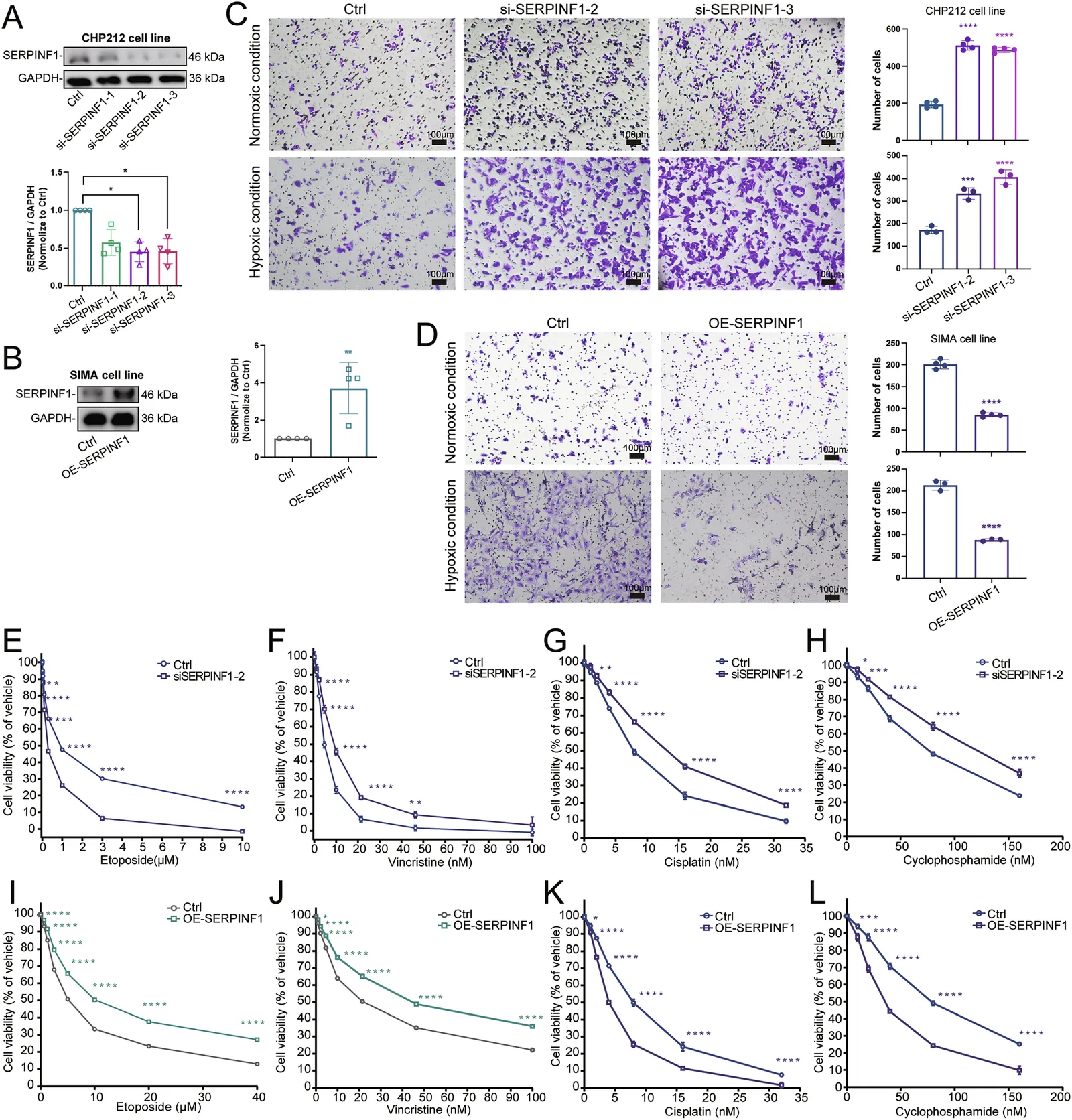
In vitro functional characterization of SERPINF1 in neuroblastoma cells. **A** Western blot analysis of SERPINF1 expression in CHP212 cells transfected with control or si-SERPINF1-1/2/3 (left), with quantification shown on the right. **B** Western blot analysis of SERPINF1 expression in SIMA cells transfected with control or SERPINF1 overexpression plasmid (OE-SERPINF1) (left), with quantification shown on the right. **C** Representative images of Transwell invasion assays (left) and quantification of invaded cells (right) in CHP212 cells following SERPINF1 knockdown (Ctrl vs. si-SERPINF1-2/3) under normoxic and hypoxic conditions. **D** Representative images of Transwell invasion assays (left) and quantification of invaded cells (right) in SIMA cells with SERPINF1 overexpression (Ctrl vs. OE-SERPINF1) under normoxic and hypoxic conditions. Cell viability curves showing the response of CHP212 cells transfected with control or si-SERPINF1-2 to increasing concentrations of etoposide (**E**), vincristine (**F**), cisplatin (**G**), and cyclophosphamide (**H**), as determined by the CCK-8 assay after 48 h of treatment. Cell viability curves showing the response of SIMA cells transfected with control or OE-SERPINF1 to increasing concentrations of etoposide (**I**), vincristine (**J**), cisplatin (**K**), and cyclophosphamide (**L**), as determined by the CCK-8 assay after 48 h of treatment.

To further explore the molecular function of SERPINF1, we performed in vitro experiments to assess its role in NB cell behavior. To account for tumor heterogeneity, we selected two NB cell lines with distinct MYCN status and endogenous SERPINF1 expression levels: CHP212 (MYCN-amplified, high SERPINF1 expression) for knockdown, and SIMA (MYCN non-amplified, low SERPINF1 expression) for overexpression (Fig. 8C). This strategy enabled us to evaluate the functional impact of SERPINF1 across different molecular contexts. Western blot analysis confirmed that si-SERPINF1-2/3 significantly reduced SERPINF1 expression in CHP212 cells (Fig. 9A), whereas OE-SERPINF1 successfully enhanced its expression in SIMA cells (Fig. 9B). Given that the NBTRP low-risk group was enriched in invasion-related pathways (Fig. 6D), we next examined the impact of SERPINF1 on cell invasiveness using Transwell assays under both normoxic and hypoxic conditions. SERPINF1 knockdown markedly increased the number of invading CHP212 cells under both conditions (Fig. 9C), whereas SERPINF1 overexpression significantly reduced the invasive capacity of the SIMA cells in both normoxic and hypoxic settings (Fig. 9D).

Given that SERPINF1 was identified as a key feature within the NBTRP model and is associated with chemotherapy-related transcriptional alterations, we further evaluated the effect of whether SERPINF1 influences cellular sensitivity to commonly used chemotherapeutic agents in NB, including etoposide, vincristine, cisplatin, and cyclophosphamide (Figs. 9E–L and S10B, C). In CHP212 cells, SERPINF1 knockdown significantly increased sensitivity to etoposide, as evidenced by reduced cell viability compared with control cells (Fig. 9E). In contrast, SERPINF1 silencing led to increased cell viability under vincristine, cisplatin, and cyclophosphamide treatment (Fig. 9F–H), indicating reduced sensitivity to these agents. Conversely, in SIMA cells, SERPINF1 overexpression resulted in increased cell viability under etoposide treatment (Fig. 9I), suggesting decreased sensitivity. For vincristine, SERPINF1 overexpression also led to increased cell viability (Fig. 9J), consistent with reduced drug sensitivity. In contrast, SERPINF1 overexpression decreased cell viability in response to cisplatin and cyclophosphamide (Fig. 9K, L), indicating enhanced sensitivity to these agents. Collectively, these results demonstrate that SERPINF1 exerts a drug-specific regulatory effect on chemotherapeutic response in NB cells. While SERPINF1 expression is associated with resistance to etoposide, it appears to enhance sensitivity to cisplatin and cyclophosphamide, and exhibits a distinct effect on vincristine response. These findings highlight a context-dependent role of SERPINF1 in chemotherapy response, supporting its potential as both a therapeutic target and a biomarker for treatment stratification in neuroblastoma.

## Discussion

Despite recent advances in understanding the neuroblastoma TME, most studies have not fully captured the treatment-induced dynamics of the stromal components [16, 17]. Here, by integrating single-cell and bulk RNA-seq data, we provide a comprehensive analysis of chemotherapy-induced alterations in adrenal NB, with a particular focus on fibroblasts. Unlike previous studies that offered limited insights into CAF remodeling [4, 18], our study systematically characterized treatment-driven changes and identified c2 SFRP2^+^ iCAFs as a dominant fibroblast subtype enriched post-chemotherapy, displaying pro-inflammatory and angiogenic signatures. Furthermore, based on transcriptional differences between pre- and post-treatment tumor cells, we developed a 6-gene prognostic model, NBTRP, that predicted overall survival across three independent patient cohorts. Among these genes, SERPINF1 has emerged as the top prognostic factor, potentially linking stromal remodeling to tumor aggressiveness and chemotherapy resistance. Collectively, our findings offer new insights into TME remodeling and suggest SERPINF1 and the NBTRP signature as promising targets for improving NB therapeutic strategies.

Previous studies have reported the presence of CAFs in NB, but their subtype heterogeneity and chemotherapy-induced plasticity remain largely uncharacterized [39, 40]. For instance, Li et al. used single-cell and spatial transcriptomics to identify a subset of senescence-associated CAFs in NB, which showed prognostic relevance in stage 4 patients [39]. Another study reported that CAFs, in cooperation with TAMs, could activate the TGF-β/IL-6 axis, establishing an immunosuppressive and tumor-promoting niche in NB [40]. In contrast to these subtype-nonspecific or pathway-centric findings, our study provides a transcriptome-level classification of NB CAF subtypes, with a specific focus on treatment-induced changes. We identified SFRP2⁺ iCAFs as the dominantly enriched subtype after chemotherapy. These cells highly expressed FBLN1 and SFRP2, and were enriched in pathways such as complement and coagulation, angiogenesis, hypoxia, and inflammatory responses. Some studies have implicated FBLN1 as a potential tumor biomarker and TME-targetable molecule in several cancers, including cervical cancer [41] and melanoma [42]. Similarly, SFRP2 has been reported to be expressed in head and neck squamous cell carcinoma [15] and ovarian cancer [43]. A recent study further demonstrated that SFRP2 is a CAF-specific gene upregulated following radio-immunotherapy in non-irradiated tumors [44]. Conditional deletion of Sfrp2 in CAFs reprogrammed the vascular–immune microenvironment and facilitated CD8⁺ T cell infiltration into distant, non-irradiated tumor sites [44]. These findings align with our observations in NB, where SFRP2⁺ iCAFs were significantly enriched in the post-chemotherapy TME, suggesting a conserved role for SFRP2-expressing fibroblasts in modulating immune exclusion and therapeutic resistance across multiple cancer types.

In addition to dissecting TME remodeling, our study established a chemotherapy-associated prognostic model for NB, termed NBTRP, which demonstrated predictive power across multiple cohorts. The 6-gene signature includes known oncogenic or TME-relevant genes such as SERPINF1. SERPINF1 encodes pigment epithelium-derived factor (PEDF) [45], a secreted glycoprotein previously associated with poor prognosis in renal clear cell carcinoma [46]. Notably, Song et al. showed that SERPINF1 promotes glioma cell proliferation and that its knockdown significantly impairs glioma growth in vitro [47]. These findings contrast with our own observations in NB, where SERPINF1 knockdown in CHP212 cells enhanced invasion, whereas SERPINF1 overexpression in SiMa cells suppressed invasive capacity, highlighting its potential role as a context-dependent functional effector in the NBTRP model.

The NBTRP model demonstrated a reliable prognostic performance for neuroblastoma across multiple independent cohorts. In comparison, Wang et al. recently developed a five-gene prognostic signature (PRGs) that achieved AUCs of 0.67 and 0.71 for 1- and 3-year survival prediction, respectively, in their validation set [48]. NBTRP outperformed PRGs at 1 year (mean AUC = 0.785) but showed a slightly lower AUC at 3 years (0.696). Notably, the PRGs model was specifically developed for high-risk, MYCN–non-amplified neuroblastoma, whereas NBTRP was trained on broader transcriptomic data, providing greater generalizability. Moreover, when compared to another XGBoost-based model developed by Yang et al., which yielded a time-independent AUC of 0.638 in the test set [49], NBTRP showed superior discriminative ability with a mean test-set AUC of 0.809. Taken together, these results highlight the competitive performance and broader applicability of the NBTRP model for stratifying patients with NB.

Tumor-associated phenotypes, including transformed cancer cells and malignant stromal populations, are increasingly being used as functional hallmarks of cancer behavior [50]. CAFs play protumorigenic roles in extracellular matrix remodeling, immunosuppressive niche, and angiogenesis [51]. This is consistent with our finding that SFRP2^+^ CAFs may promote angiogenesis in NB through the CCL2–ACKR1 and CXCL2/3/8–ACKR1 signaling axes. In addition, NB is characterized by a profoundly immunosuppressive TME, which presents challenges for immunotherapy [52]. In our study, the composition of immune infiltration differed significantly across NBTRP-defined risk groups, with high-risk patients showing reduced infiltration of cytotoxic lymphocytes and T cells. This suggests that NBTRP may serve as a stratification tool for identifying patients who are more likely to benefit from immune-based therapies. Furthermore, several chemotherapeutic agents currently used or under clinical investigation for NB and other solid tumors, such as doxorubicin [53], etoposide [54], gemcitabine [55], and vinblastine [56], were predicted by the NBTRP model to exhibit greater efficacy in high-risk patients. Consistently, our experimental data revealed that modulation of SERPINF1 expression exerted drug-specific responses in neuroblastoma cells, conferring resistance to vincristine and etoposide, while enhancing sensitivity to cisplatin and cyclophosphamide. This dichotomous behavior suggests that SERPINF1 plays a multifaceted role in mediating both tumor invasiveness and chemotherapy responsiveness, reflecting the complex adaptive nature of the neuroblastoma microenvironment. Collectively, these findings highlight NBTRP, particularly its key component SERPINF1, as a novel prognostic framework that captures chemotherapy-associated transcriptional dynamics, enables robust patient stratification, and offers actionable insights into precision-guided therapeutic strategies in neuroblastoma.

This study has several limitations. First, although spatial transcriptomic and immunofluorescence analyses supported the characterization of SFRP2+ iCAFs, these validations were limited in sample size. Second, although the NBTRP model showed robust performance across multiple cohorts, clinical heterogeneity, including differences in treatment regimens and sequencing platforms, may affect its generalizability. Third, functional validation was focused on SERPINF1, and further mechanistic studies are required to elucidate its downstream pathways. Finally, although paired pre- and post-treatment analyses provided insights into chemotherapy-associated TME remodeling, the limited number of matched samples still constrains statistical power, and prospective longitudinal studies will be required to validate NBTRP-guided therapeutic strategies.

## Conclusion

In summary, this study identified SFRP2⁺ iCAFs as a key stromal subtype enriched after chemotherapy in adrenal neuroblastoma, with transcriptional and ligand–receptor features suggestive of angiogenic, immunomodulatory, and tumor-promoting functions. These findings expand our understanding of treatment-induced TME remodeling and highlight CAFs as potential therapeutic targets in the post-chemotherapy context. Moreover, we developed NBTRP, a chemotherapy-associated 6-gene prognostic signature that effectively stratifies patients according to survival outcomes and therapeutic susceptibilities. SERPINF1 has emerged as a key prognostic and functional effector gene that modulates tumor invasiveness and influences drug responses in a cell type– and context– dependent manner, suggesting that its impact on chemosensitivity may vary according to the molecular state of the tumor. Together, these findings provide a mechanistic and translational framework linking stromal reprogramming with tumor adaptation and treatment response, offering new directions for precision prognostication and individualized therapy for neuroblastoma.

## Supplementary information

Supplementary figures

Supplementary tables

Original Western blots

Reproducibility checklist

## Data Availability

The scRNA-seq data can be accessed from GEO under accession numbers GSE137804 [17] and GSE218003 [18]. Bulk RNA-seq and clinical information can be obtained from the GSE49710, E-MTAB-8248, and TARGET-NB datasets. In addition, snRNA-seq data are available from Yu et al. [16], and spatial transcriptomic data can be obtained from Siaw, JT et al. [19]. For any other reasonable requests, please contact the corresponding author.

## Code availability

The NBTRP model is available at https://github.com/JunekureWu/NBTRP.
