# Supplementary material for: SFRP2+ fibroblasts orchestrate post-chemotherapy remodeling and define a tumor-driven prognostic program, NBTRP, in adrenal neuroblastoma
Source: Cell Death Dis. 2026 Aug 1;17(1):915. doi: 10.1038/s41419-026-09132-y (PMC13720200; doi:10.1038/s41419-026-09132-y)
Supplement: Supplementary file 1 [file 41419_2026_9132_MOESM1_ESM.pdf]

Supplementary figures S1 to S10.

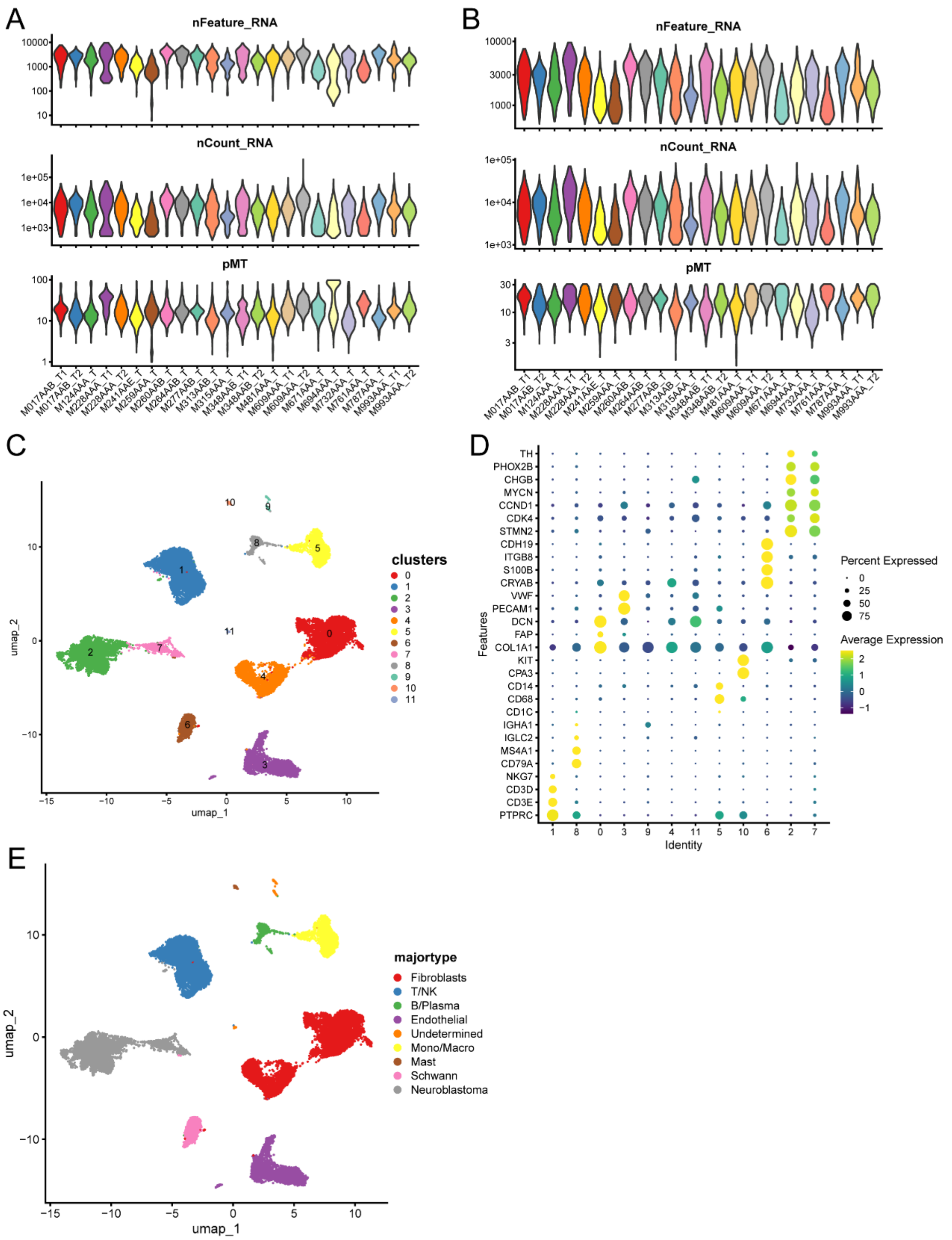

Figure S1. Quality control and cell type annotation of the neuroblastoma chemotherapy cohort (GSE218003)

(A–B) Distributions of nFeature_RNA, nCount_RNA, and percent.mt across 24 samples before (A) and after (B) quality control filtering. Each color represents a different sample.

(C) UMAP visualization showing 12 clusters identified by Seurat after unsupervised clustering.

(D) Expression of representative marker genes across the 12 clusters.
(E) Cell type annotation results, identifying 8 major cell types and 1 undetermined cluster.

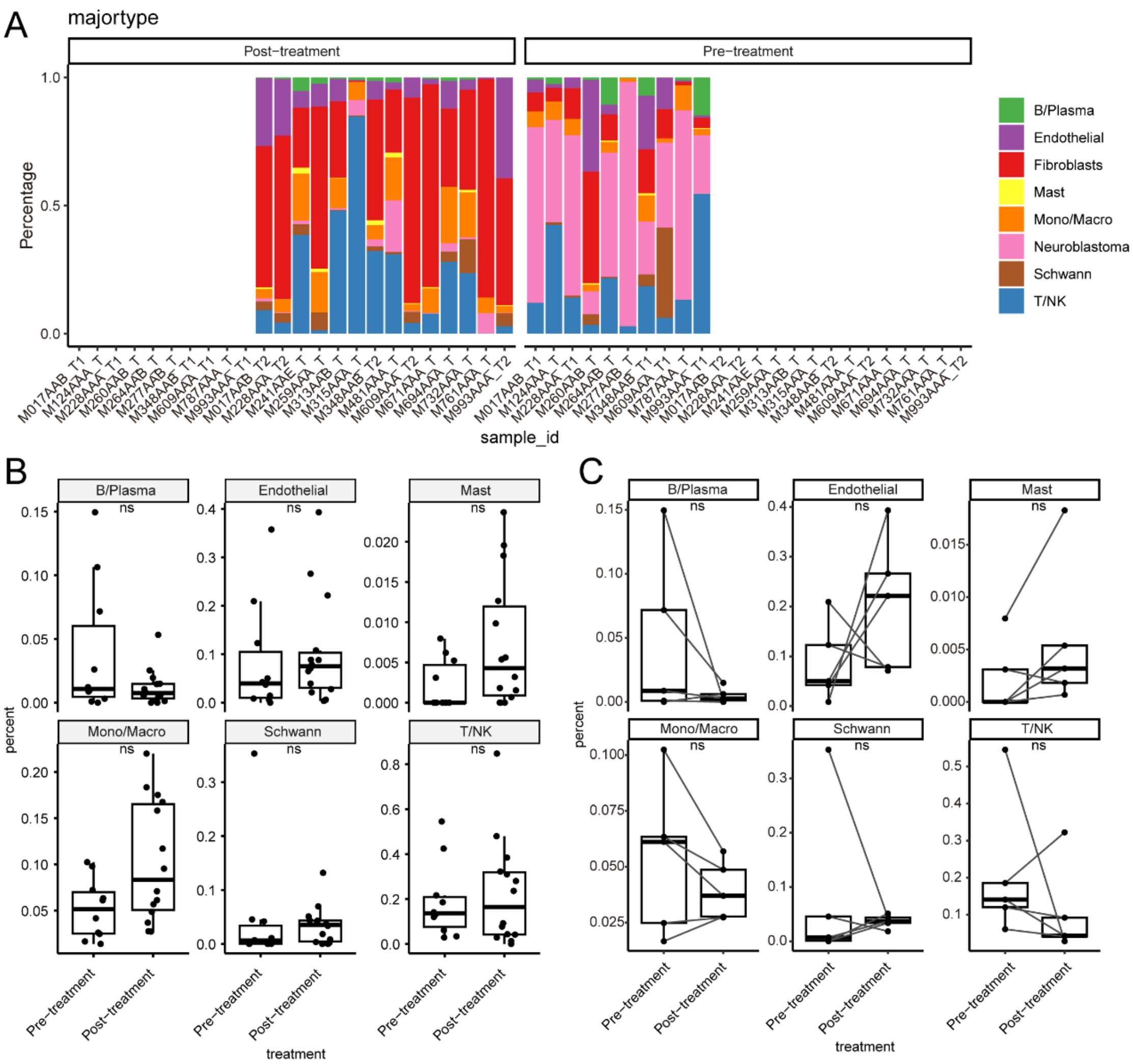

Figure S2. Proportion analysis of major cell types between pre-treatment and post-treatment groups

(A) Bar plot showing the proportions of eight major cell types across all 24 samples (10 pre-treatment and 14 post-treatment).

(B) Box plots comparing the proportions of six major cell types—excluding fibroblasts and NB cells—between pre-treatment and post-treatment groups using the Wilcoxon rank-sum test.

(C) Box plots comparing the same six cell types between matched pre-treatment and post-treatment samples using the paired t-test.

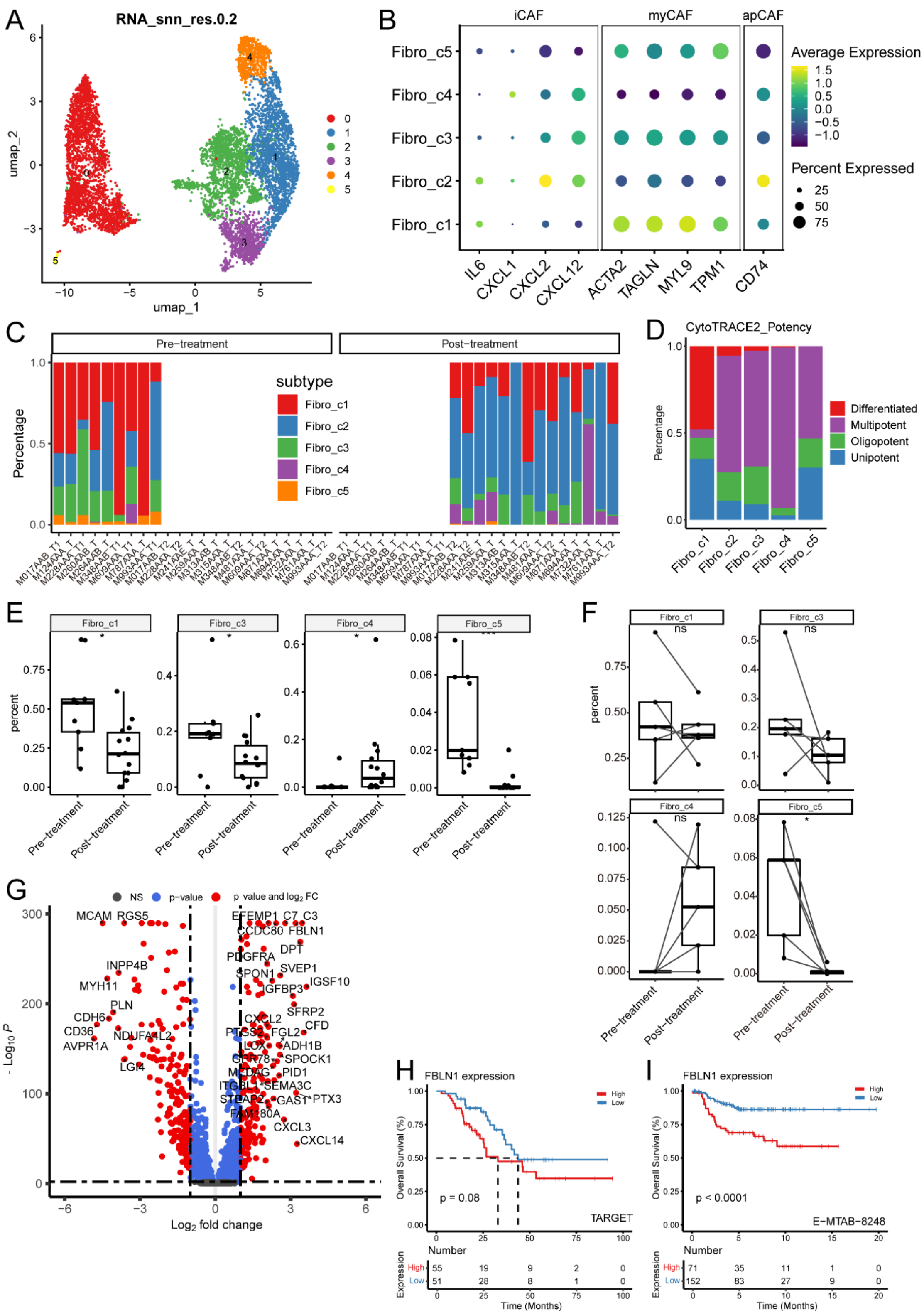

Figure S3. Subtype characterization and prognostic relevance of fibroblast subpopulations

(A) UMAP plot showing six fibroblast clusters identified using Seurat.

(B) Expression of canonical CAF marker genes for iCAF, myoCAF, and apCAF across five fibroblast subtypes (Fibro_c1–c5), after merging clusters 1 and 2 into a combined cluster (c2).

(C) Bar plot showing the proportions of the five CAF subtypes across 10 pre-treatment and 14 post-treatment samples.
(D) Bar plot comparing the distribution of four CytoTRACE2-inferred potency states across the five CAF subtypes.
(E) Box plots comparing the proportions of four major CAF subtypes between pre-treatment and post-treatment samples using the Wilcoxon rank-sum test.
(F) Paired comparison of the same four CAF subtypes between matched pre-treatment and post-treatment samples using the paired t-test.
(G) Volcano plot showing differentially expressed genes (DEGs) in the $SFRP2^{+}$ iCAF (c2) cluster.
(H–I) Kaplan–Meier survival curves for FBLN1 expression in the TARGET (H) and E-MTAB-8248 (I) cohorts. P-values were calculated using the log-rank test.

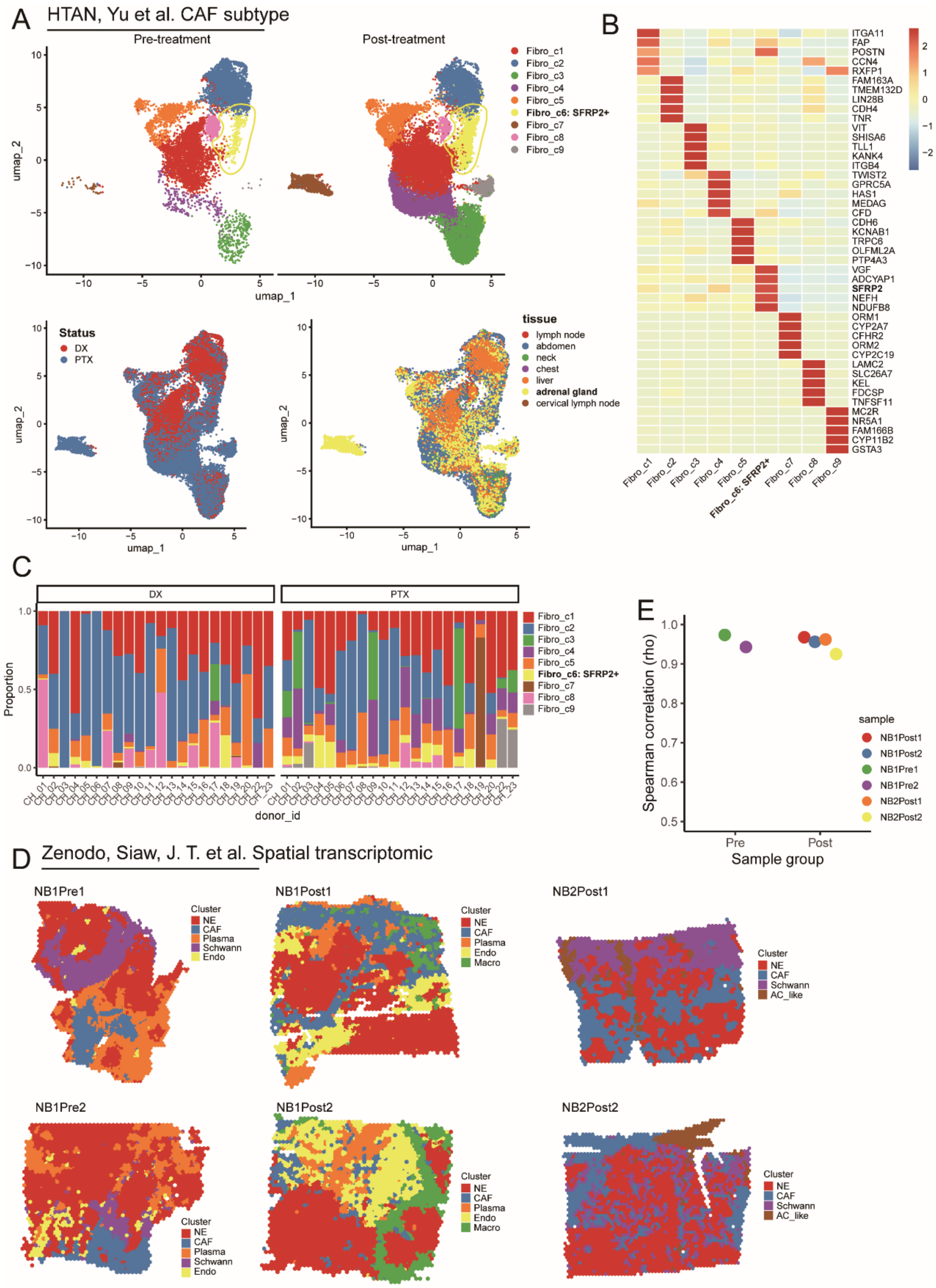

Figure S4. Analysis of Yu et al. snRNA-seq cohort and Siaw, J.T. et al. spatial transcriptomic dataset.

(A) UMAP plot showing nine fibroblast clusters identified using Seurat, along with the treatment status and tissue original.

(B) Heatmap of the top 5 differentially expressed genes across the nine CAF subtypes.

(C) Bar plot showing the proportions of the nine CAF subtypes across 22 paired pre- and post-treatment samples.

(D) Spatial distributions of six human high-risk NB samples.

(E) Spatial correlations between SRFP2⁺FBLN1⁺ cells and CAFs.

DX, pre-treatment diagnosis; PTX, post-induction chemotherapy. Pre, treatment-naïve; Post, chemotherapy treated.

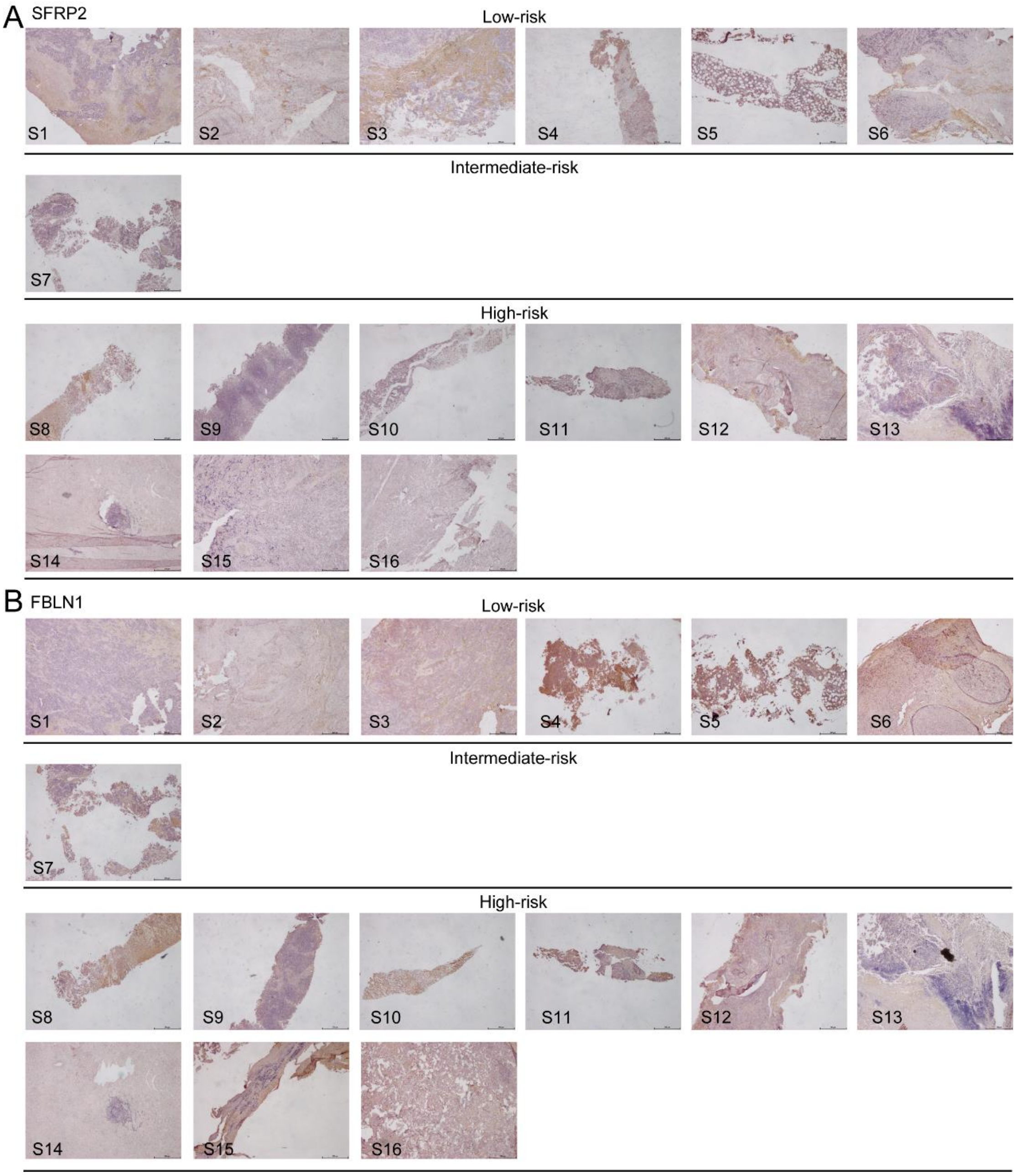

Figure S5. IHC staining of human neuroblastoma samples

(A-B) Representative IHC staining of SFRP2 (A) and FBLN1 (B) in human NB samples across different clinical risk stratifications (low-risk, n=6; intermediate-risk, n=1; and high-risk, n=9 groups).

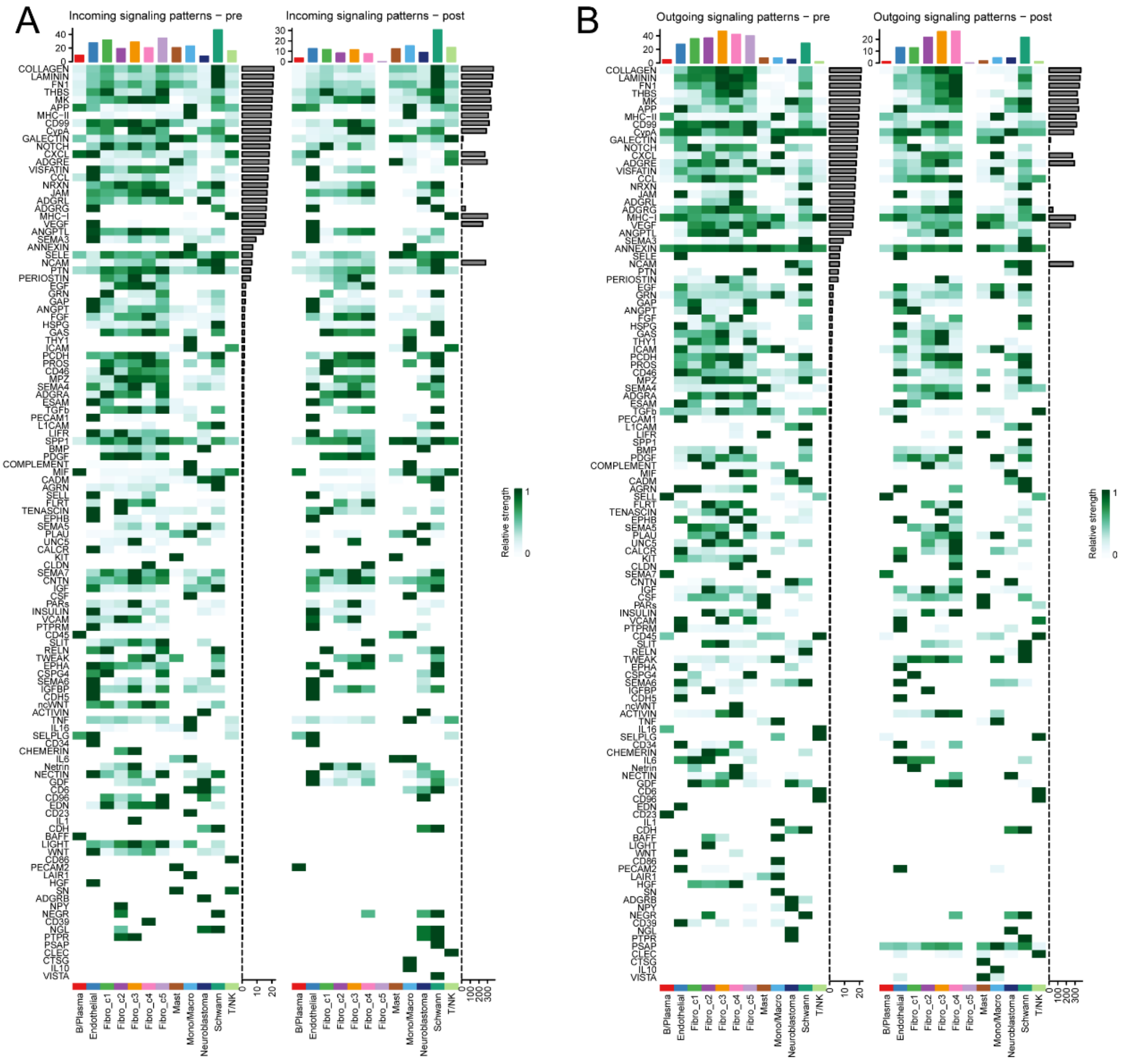

Figure S6. Global comparison of cell–cell communication strength before and after chemotherapy

(A) Heatmap showing the incoming signaling strength of all ligand–receptor pairs across cell types in pre-treatment (left) and post-treatment (right) samples.

(B) Heatmap showing the outgoing signaling strength of all ligand–receptor pairs across cell types in pre-treatment (left) and post-treatment (right) samples.

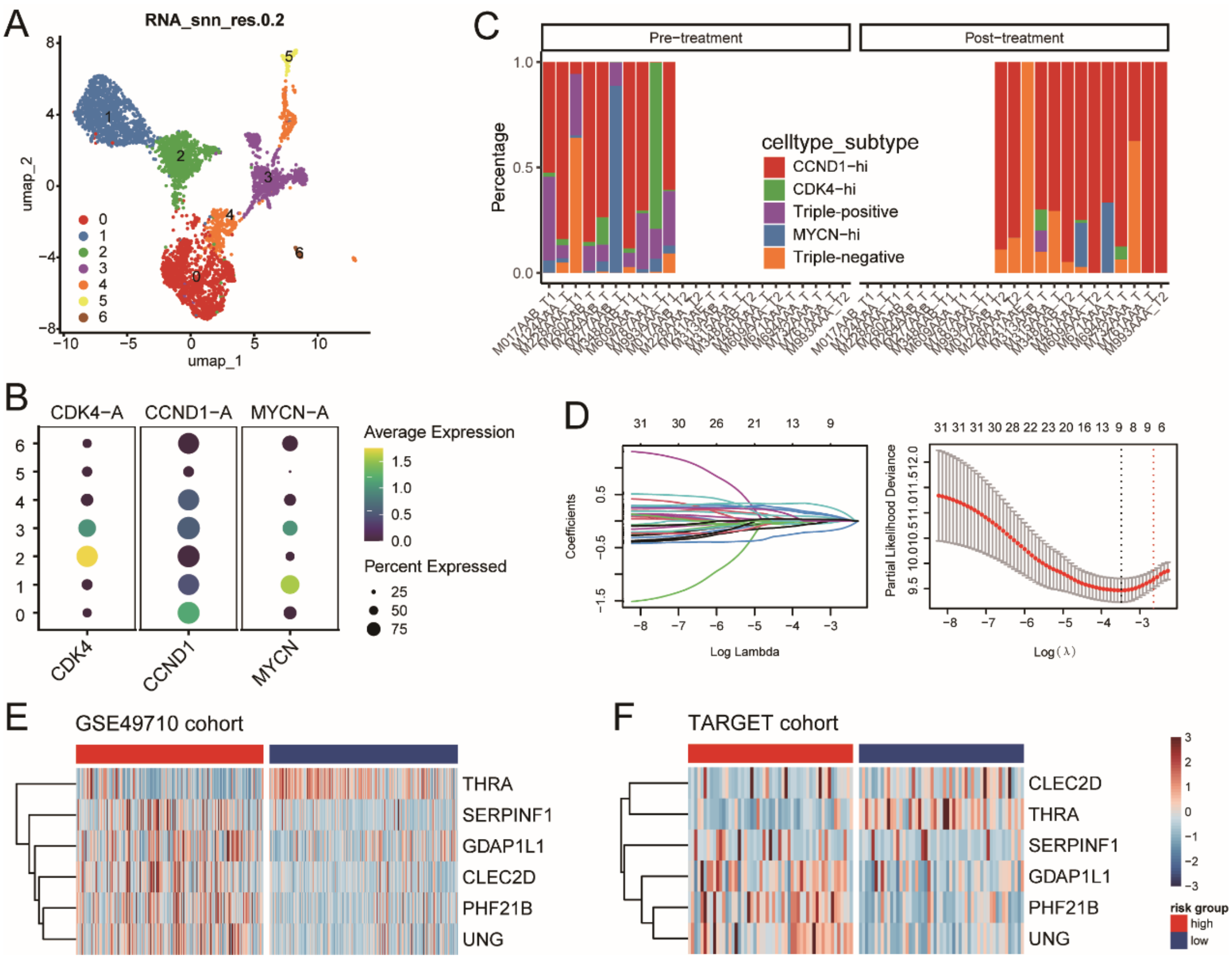

Figure S7. NB cell subpopulation profiling and model construction process.

(A) UMAP plot showing seven neuroblastoma (NB) clusters identified using Seurat.

(B) Expression of representative NB marker genes (CDK4, CCND1, and MYCN) across the seven clusters shown in (A).

(C) Bar plot showing the proportions of five NB subtypes across 10 pre-treatment and 14 post-treatment samples.

(D) LASSO Cox regression model construction using 32 candidate genes. Coefficient trajectories of all features over log(lambda) (left), and 10-fold cross-validation deviance curve (right).

(E–F) Heatmaps showing expression patterns of the six NBTRP signature genes in the GSE49710 (E) and TARGET (F) cohorts, stratified by high- and low-risk groups.

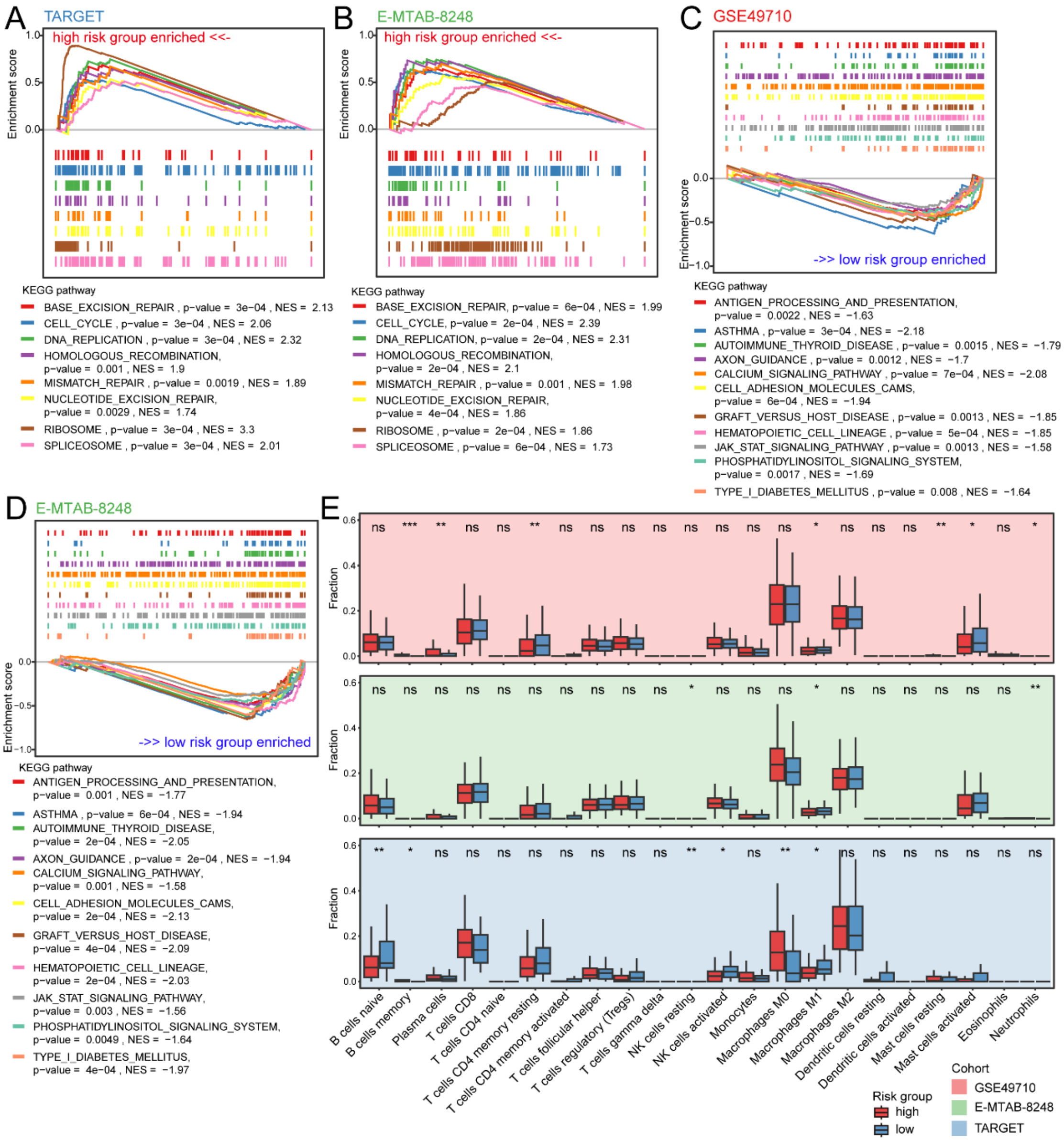

Figure S8. Functional enrichment and cell infiltration associated with NBTRP risk groups across independent cohorts

(A–B) GSEA plots of the eight overlapping KEGG pathways enriched in the NBTRP high-risk group, as identified in Figure 5A, shown for the TARGET (A) and E-MTAB-8248 (B) cohorts.

(C–D) GSEA plots of the eleven overlapping KEGG pathways enriched in the NBTRP low-risk group, as identified in Figure 5C, shown for the GSE49710 (C) and E-MTAB-8248 (D) cohorts.

(E) CIBERSORT-estimated immune cell infiltration fractions in NBTRP high- and low-risk groups across the GSE49710, TARGET, and E-MTAB-8248 cohorts.

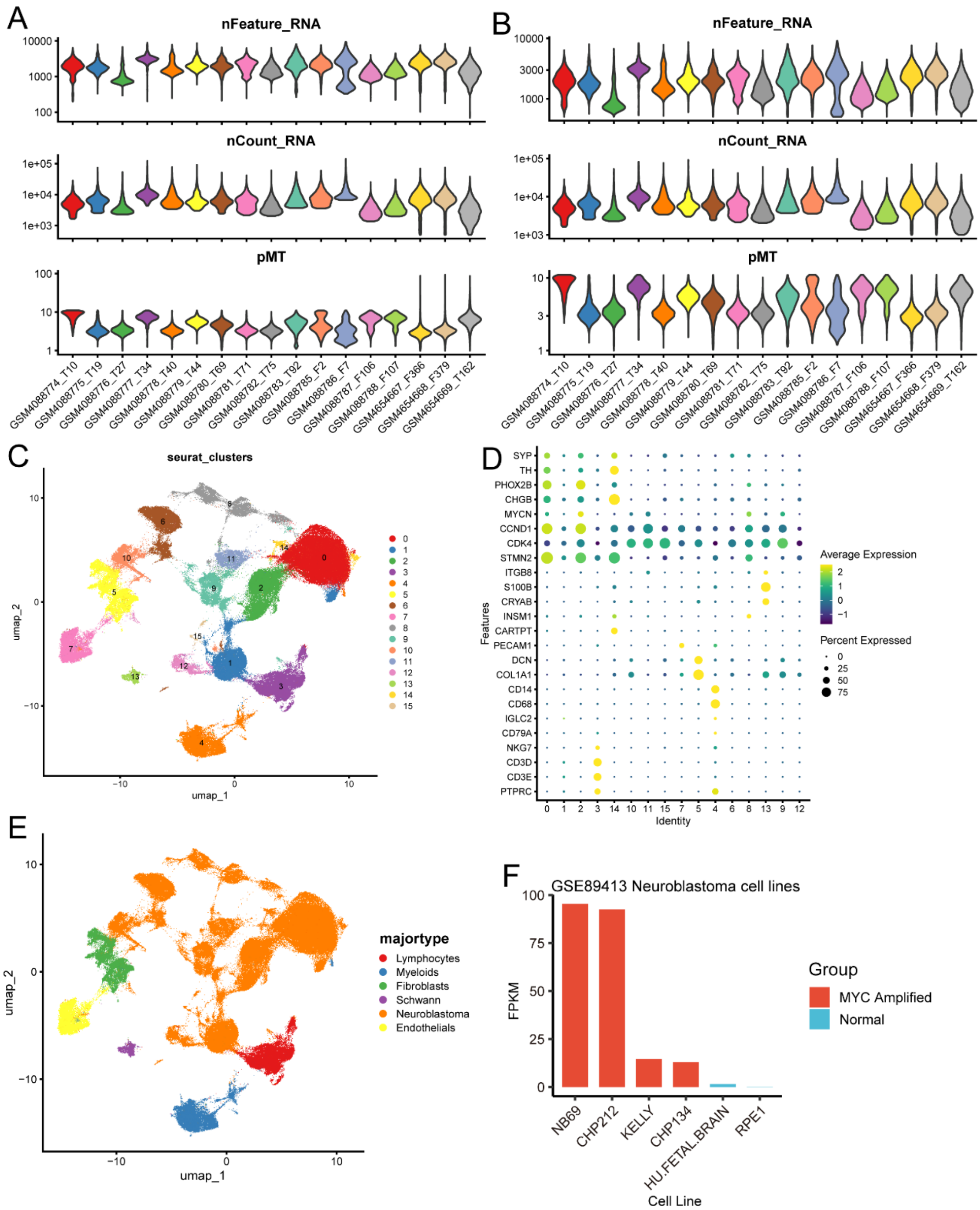

Figure S9. Preprocessing of the GSE137804 scRNA-seq dataset and SERPINF1 expression in NB cell lines

(A – B) Distributions of nFeature_RNA, nCount_RNA, and percent.mt across 17 samples before (A) and after (B) quality control filtering. Each color represents an individual sample.

(C) UMAP visualization showing 16 cell clusters identified using Seurat after unsupervised clustering.

(D) Expression patterns of representative marker genes across the 16 clusters shown in (C).

(E) Cell type annotation results identifying six major cell types across the dataset.

(F) Expression levels of SERPINF1 (FPKM values) from the GSE89413 dataset across

four NB cell lines and two controls: human fetal brain tissue and RPE1 cells.

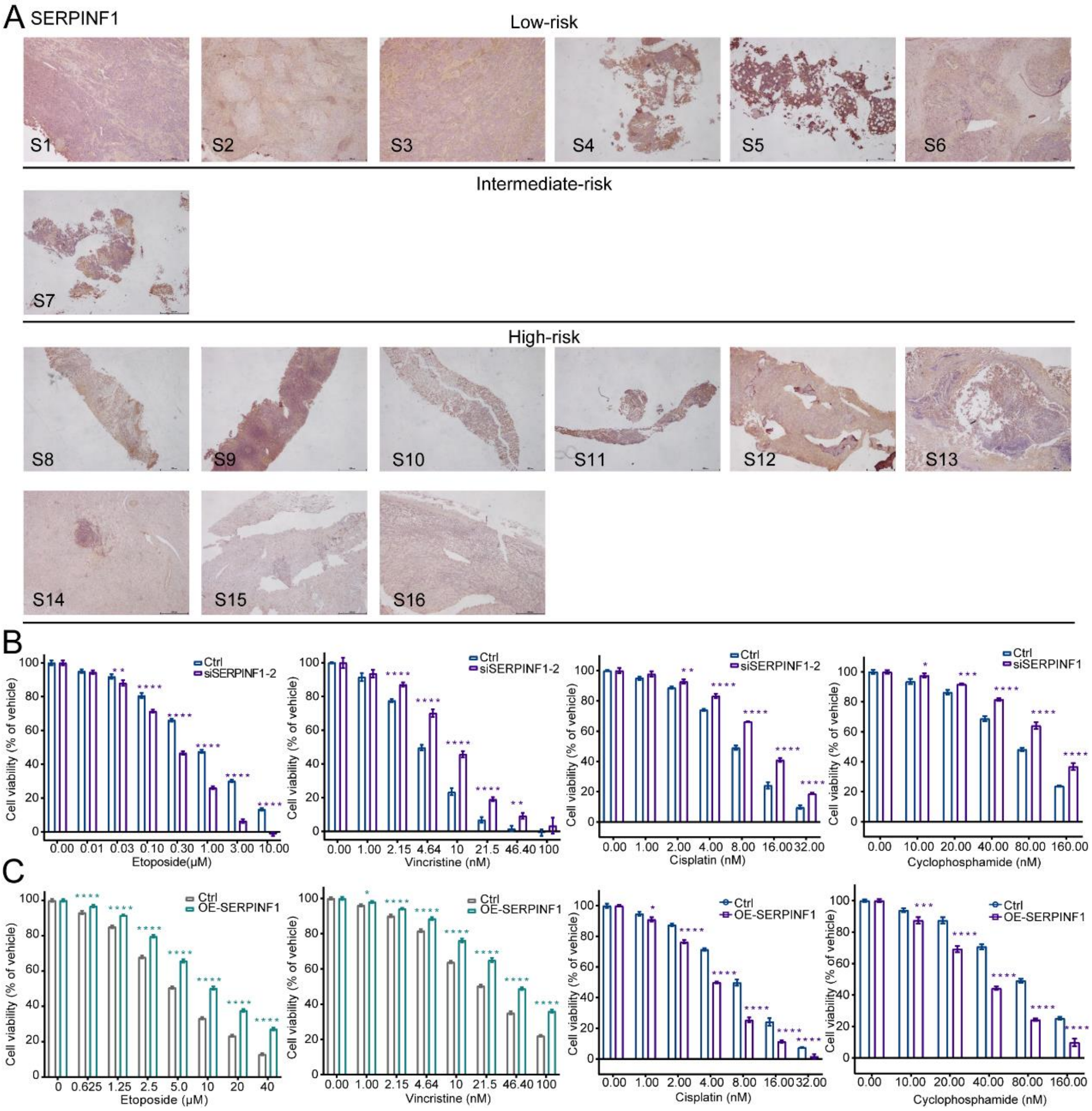

Figure S10. IHC staining of human neuroblastoma and in vitro functional characterization of SERPINF1 in neuroblastoma cells.

(A) Representative IHC staining of SERPINF1 in human NB samples across different clinical risk stratifications (low-risk, intermediate-risk, and high-risk groups).

(B) Cell viability bar plots showing the response of CHP212 cells transfected with control or si-SERPINF1-2 to increasing concentrations of etoposide, vincristine, cisplatin, and cyclophosphamide, as determined by the CCK-8 assay after 48 h of treatment.

(C) Cell viability bar plots showing the response of SIMA cells transfected with control or OE-SERPINF1 to increasing concentrations of etoposide, vincristine, cisplatin, and cyclophosphamide, as determined by the CCK-8 assay after 48 h of treatment.
