# Supplementary material for: SFRP2+ fibroblasts orchestrate post-chemotherapy remodeling and define a tumor-driven prognostic program, NBTRP, in adrenal neuroblastoma
Source: Cell Death Dis. 2026 Aug 1;17(1):915. doi: 10.1038/s41419-026-09132-y (PMC13720200; doi:10.1038/s41419-026-09132-y)

Figure 9A CHP212 cell line

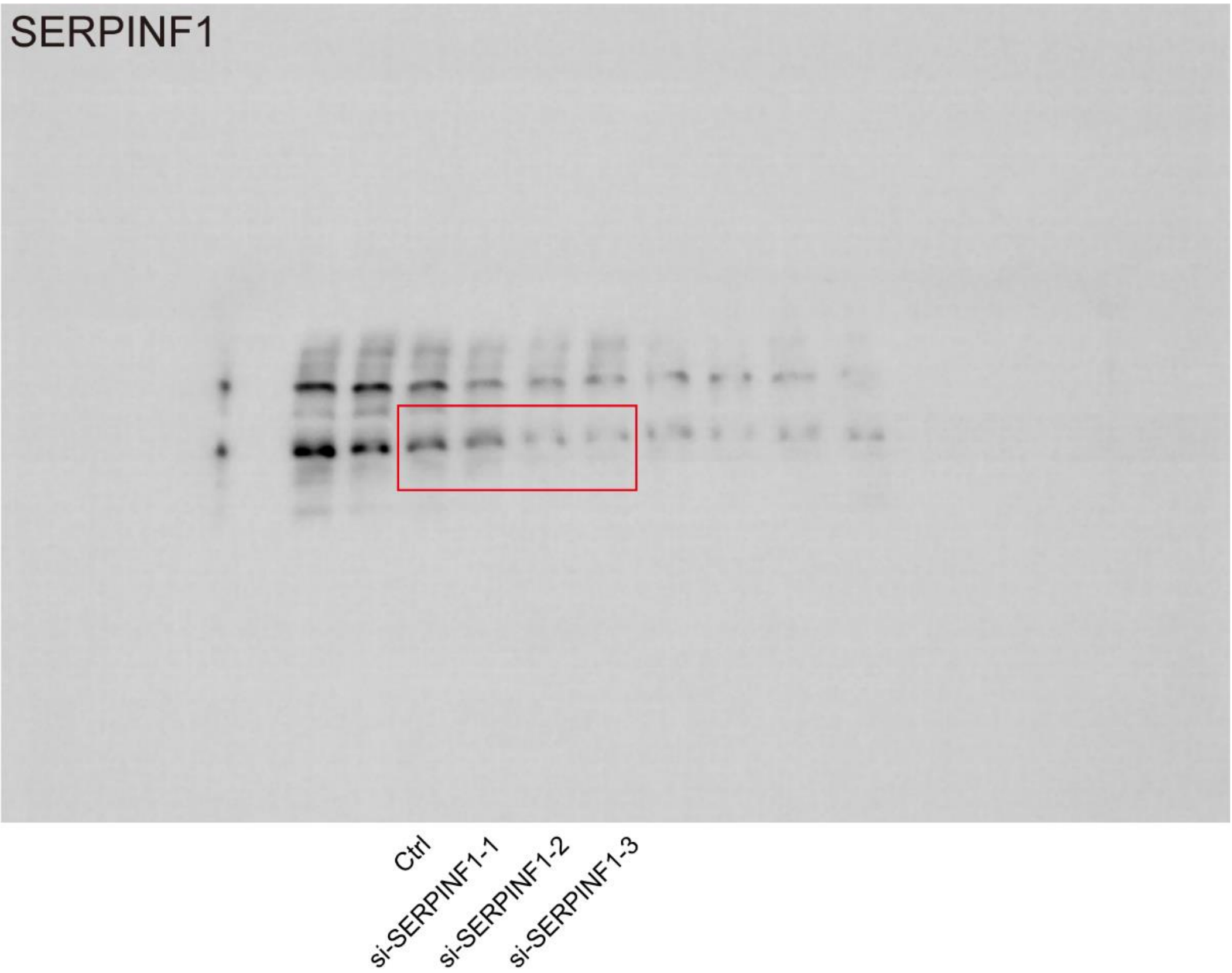

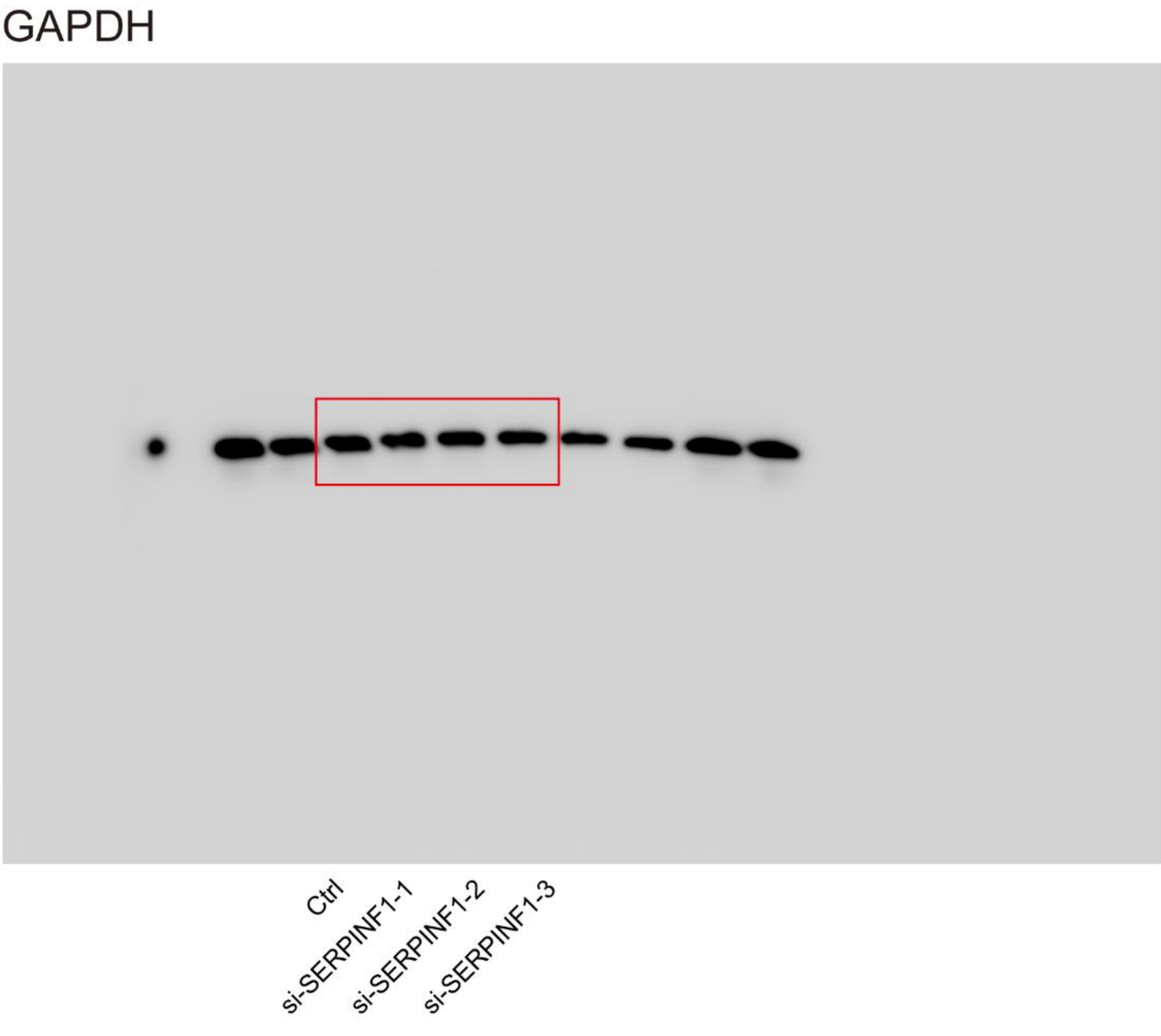

Figure 9B SIMA cell line

SERPINF1

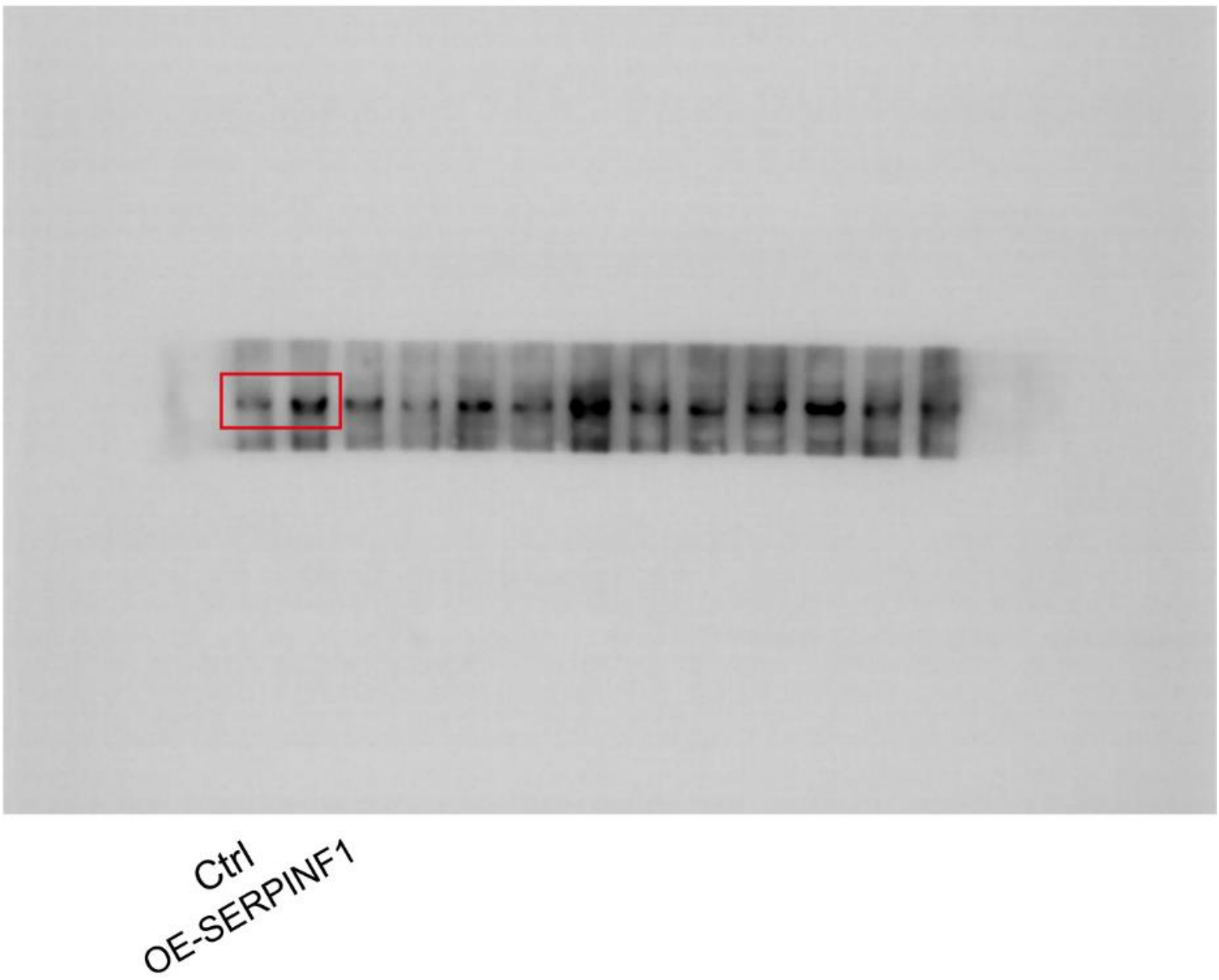

GAPDH

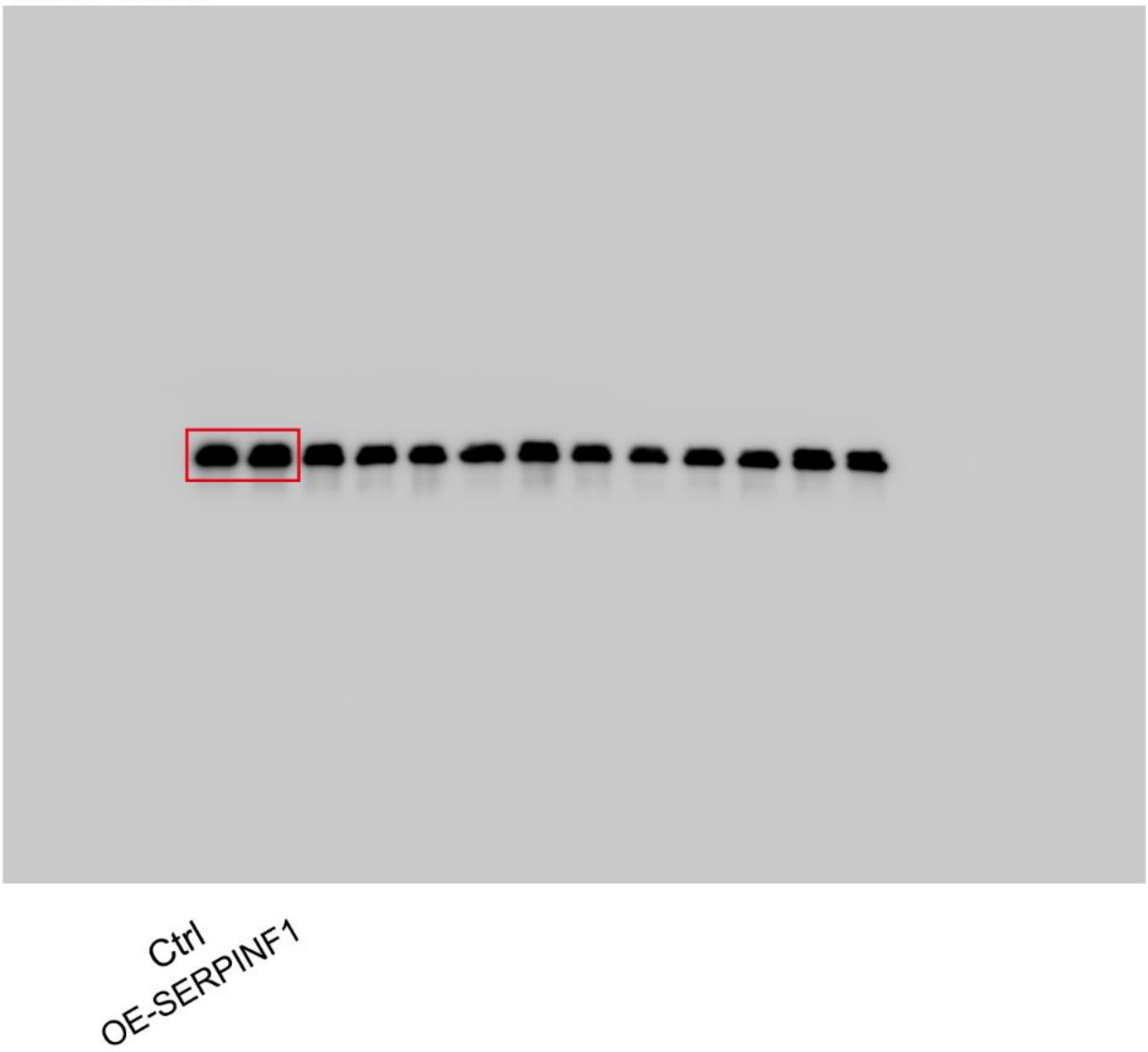

Supplement: Supplementary file 2 [file 41419_2026_9132_MOESM3_ESM.pdf]
